# The Tumor Microenvironment as a Driving Force of Breast Cancer Stem Cell Plasticity

**DOI:** 10.3390/cancers12123863

**Published:** 2020-12-21

**Authors:** Flavia Fico, Albert Santamaria-Martínez

**Affiliations:** Swiss Institute for Experimental Cancer Research, École Polytechnique Fédérale de Lausanne, CH-1015 Lausanne, Switzerland; flaviafc@hotmail.it

**Keywords:** tumor microenvironment, cancer stem cells, breast cancer, extracellular matrix, immune cells, stromal cells

## Abstract

**Simple Summary:**

Breast cancer stem cells are a subset of transformed cells that sustain tumor growth and can metastasize to secondary organs. Since metastasis accounts for most cancer deaths, it is of paramount importance to understand the cellular and molecular mechanisms that regulate this subgroup of cells. The tumor microenvironment (TME) is the habitat in which transformed cells evolve, and it is composed by many different cell types and the extracellular matrix (ECM). A body of evidence strongly indicates that microenvironmental cues modulate stemness in breast cancer, and that the coevolution of the TME and cancer stem cells determine the fate of breast tumors. In this review, we summarize the studies providing links between the TME and the breast cancer stem cell phenotype and we discuss their specific interactions with immune cell subsets, stromal cells, and the ECM.

**Abstract:**

Tumor progression involves the co-evolution of transformed cells and the milieu in which they live and expand. Breast cancer stem cells (BCSCs) are a specialized subset of cells that sustain tumor growth and drive metastatic colonization. However, the cellular hierarchy in breast tumors is rather plastic, and the capacity to transition from one cell state to another depends not only on the intrinsic properties of transformed cells, but also on the interplay with their niches. It has become evident that the tumor microenvironment (TME) is a major player in regulating the BCSC phenotype and metastasis. The complexity of the TME is reflected in its number of players and in the interactions that they establish with each other. Multiple types of immune cells, stromal cells, and the extracellular matrix (ECM) form an intricate communication network with cancer cells, exert a highly selective pressure on the tumor, and provide supportive niches for BCSC expansion. A better understanding of the mechanisms regulating these interactions is crucial to develop strategies aimed at interfering with key BCSC niche factors, which may help reducing tumor heterogeneity and impair metastasis.

## 1. Introduction

The incidence of breast cancer (BC) has been raising over the last years, with an annual increase of 3.1% and significant variations between countries: North America, Australia, New Zealand, and Northern and Western Europe show the highest incidence compared to lower income regions, with a distribution pattern that reflects the availability of detection methods and the risk factors associated to the disease [1]. Conversely, BC mortality rate is higher in low-income countries due to the limited access to treatments and the late stage at diagnosis [2]. At present, patients with early-stage tumors, which account for 70–80% of the total cases, are successfully treated. Advanced metastatic BC (20–30% of the cases) remains incurable, and the available treatments are meant to slow its progression and to relieve the symptoms derived from the disease [3]. Overall, the 5-year survival rate is around 99% for localized BC, 86% for regional disease, and 27% for metastatic BC (BC stage IV) [4]. Around 5–10% of BC cases are hereditary, 85% of which are related to mutations in the two high penetrance tumor suppressor genes *BRCA1* and *BRCA2* [5], whereas the majority of breast tumors are sporadic and due to somatic, genetic, and epigenetic alterations acquired during life [6]. Non-hereditary risk factors include age, body mass index, sex, density of the breast, early menarche, age at first birth, late menopause, alcohol consumption, exposure to radiation, and smoking habits.

The evolutionary dynamics in some tumor types, such as BC, are subject to hierarchical systems in which cancer stem cells (CSCs) sustain tumor growth and can colonize secondary organs. Typically, BC stem cells (BCSCs) are identified and isolated by fluorescence-activated cell sorting (FACS); the most common isolation strategies for human BC cells are the antibody staining of the cell surface markers CD24 and CD44, and the evaluation of aldehyde dehydrogenase (ALDH) activity [7], with both the Lin^−^CD24^−/low^CD44^+/high^ and the ALDH^high^ populations in breast tumors being enriched for CSCs. Tumor initiation potential—BCSC’s most distinctive feature—is tested in limiting dilution assays (primary tumors) or in metastasis assays (secondary tumors) in vivo. Moreover, the capacity to form spheres in vitro is generally used as a surrogate marker for CSC. The importance of CSC biology to the clinic is emphasized by numerous studies showing the essential role of these particular cells in metastasis, chemoresistance, and tumor relapse [8]. In the classical CSC model, a subset of cancer cells with stem cell properties can self-renew and also give rise to lineages with various degrees of differentiation. Conventionally, differentiated cells cannot revert to CSCs, since this capacity is exclusive of pluripotent cells. However, tumors are heterogeneous dynamic systems in constant evolution, and their progression is determined by the interactions between cancerous cells and the tumor microenvironment (TME), which is composed of other non-transformed cell types, such as stromal and immune cells, and the extracellular matrix (ECM). In recent years, in line with the plastic nature of tumors, the idea of a unidirectional system has been challenged by results showing that the network is strongly dependent on microenvironmental signals and on the interplay between different cell types in the tumor [8]. Thus, the complexity of the evolution of CSC-driven tumors is greater than previously thought, since in the new CSC model, non-CSCs can reacquire the CSC phenotype and CSCs can become non-CSCs depending on signals from the niche. In this manuscript, we will review the current knowledge on how components of the TME regulate the BCSC phenotype and plasticity in the BCSC niche.

## 2. Phenotypic Heterogeneity and Breast Cancer Molecular Subtypes

BC is a heterogeneous disease, and this heterogeneity is reflected in the CSC phenotypic and quantitative differences that are found between BC subtypes [9]. Likewise, it has been suggested that poorly differentiated tumors contain higher numbers of CSCs [10]. The classification of BC was originally based on histological features such as type, grade, and stage of the tumor, but the advent of microarray technology allowed researchers to classify breast tumors according to their molecular profiles [11]. Gene expression analysis led to the identification of five different subtypes of BC: luminal-like (comprising luminal subtypes A and B), HER2+, basal, and normal-like tumors. In addition, a sixth subtype was identified years later and named claudin-low, denoting the low expression of genes related to tight junctions and to cell-cell adhesion found in this group of tumors [12]. In the clinic, diagnosis and treatment decisions are still based on the immunohistochemistry markers estrogen receptor (ESR1), progesterone receptor (PGR), epidermal growth factor receptor 2 (ERBB2, also known as HER2), and the proliferation marker Ki67. However, the molecular features of tumors are known to determine their prognosis and response to therapy, and distinct gene signatures are currently used in genomic tests [13]. Luminal A tumors respond well to hormonal anti-estrogen therapy, but poorly to chemotherapy. Luminal B tumors are of a higher grade, have worse prognosis, and may benefit from combined therapies. In keeping with their more differentiated status, luminal-like tumors typically present an epithelial phenotype and contain low percentages of both CD44^+^/CD24^−^ CSCs and ALDH1^+^ CSCs. The latter represent a small fraction of CD44^+^/CD24^−^ cells and are therefore considered to be enriched for a different population of CSCs [14]. Accordingly, luminal A tumors have the lowest rates of metastatic spread compared to all the other subtypes [15,16,17]. Phenotypically, HER2+ tumors are also mainly epithelial, but they contain higher frequencies of ALDH1^+^ BCSCs [18]. Basal-like, and particularly claudin-low tumors, are highly enriched in both CD44^+^/CD24^−^ cells and ALDH1^+^ cells [19,20]. In addition, claudin-low tumors display epithelial-to-mesenchymal transition (EMT) signatures and a mesenchymal phenotype, and are associated with poor prognosis. 

BCSC plasticity is exemplified by the capacity to bidirectionally switch between epithelial and mesenchymal states [21]. This conversion is achieved through the EMT, which is a bidirectional, reversible process. For instance, upon metastatic colonization, CSCs undergo mesenchymal-to-epithelial transition (MET) in order to regain epithelial features and higher proliferative rates [22]. EMT is now widely regarded as a key mechanism that produces cancer cells with stem cell traits and enhanced metastatic ability [23], although the degree of complexity of this process is higher than originally thought [24,25,26,27,28]. In addition, it is worth noting that epithelial-like CSCs and mesenchymal-like CSCs may possess different abilities to initiate tumors in the primary or the secondary site [29], and further data indicate that the expression of EMT-related genes does not guarantee an enrichment in tumor-initiating cells [30].

## 3. The Tumor Microenvironment and the Breast Cancer Stem Cell Phenotype

The phenotypic plasticity of breast tumors is tightly regulated by cues from the TME, a complex milieu where transformed cells evolve among a myriad of signals that impact their behavior and ultimately their ecological fitness. It consists of transformed cells and the surrounding normal tissue, as well as immune cells, cancer-associated fibroblasts (CAFs), endothelial cells, and the ECM [31]. As tumors evolve, the role of each component of the TME changes, sometimes with opposing effects. These alterations are often the consequence of an adaptive process, during which cancer cells take advantage of and hijack certain elements of the TME in order to grow. 

### 3.1. Myeloid Cells

Myeloid cells arise from a common hematopoietic progenitor and comprise monocytes and macrophages, myeloid dendritic cells, neutrophils, eosinophils, basophils, mast cells, erythrocytes, and megakaryocytes (which give rise to platelets). A number of groups have shown that myeloid cells play an important role in regulating the BCSC phenotype. For instance, myeloid-derived suppressor cells, a heterogeneous group of immune cells with immune inhibitory functions, sustain BCSCs through the secretion of interleukin-6 (IL-6) and nitric oxide (NO), and activation of the STAT3 and Notch pathways [32]. However, most studies have focused on the role of tumor-associated macrophages (TAMs), since it is one of the most abundant immune cell populations in breast tumors. The importance of this subset of cells in BC progression is underlined by a number of studies showing that the degree of TAM infiltration in mammary tumors is strongly associated with tumor progression, overall survival, and relapse-free survival [33,34,35,36,37,38,39,40,41,42]. 

#### 3.1.1. Macrophages

Macrophages are classified into non-activated, classically activated (M1), and alternatively activated (M2) macrophages. M1 macrophages are proinflammatory, while M2 macrophages, which have been subclassified into different subgroups depending on the activation mechanism (M2a, M2b, M2c, and M2d), promote tumor progression and angiogenesis, and possess immunosuppressive features [43]. However, it is worth noting that current data suggest that this classification may not reflect the biological heterogeneity of macrophages, which defies the classical M1-M2 binary classification [44].

The role of macrophages in the mammary gland stem cell niche was first suggested by experiments showing that their ablation led to a significant decrease in the repopulating ability of mammary gland stem cells (MaSCs) [45]. Further work has shown that there is a cross-talk between macrophages and MaSCs, in which the MaSCs-dependent activation of Notch signaling in macrophages triggers the expression of Wnt ligands that in turn act on MaSCs [46]. In BC, the depletion of macrophages was also shown to reduce side population (SP) CSCs and to decrease tumorigenicity and metastasis in a mouse model of mammary gland tumorigenesis [47]. The authors found that TAM-secreted epidermal growth factor (EGF) activated the expression of the BCSC promoting factor Sox2 through a STAT3-dependent mechanism, driving CSC expansion [47,48]. Consistent with the results showing that macrophage ablation leads to CSC depletion, mixing TAMs with CSCs increased tumor initiation, sphere formation, the metastatic index, and tumor-free survival, which further supports the role of macrophages in CSC biology [49]. The coculture of monocytes and CD90^hi^CD24^−^ HMLE-Ras (HMLER90hi) CSCs resulted in a rapid induction of IL-6 and IL-8, which promoted CSC self-renewal in both an autocrine and a paracrine manner [49,50,51]. It is worth noting that cancer cells can express factors such as multiple copies in T-cell malignancy 1 (MCT-1) or yes-associated protein 1 (YAP), which activate IL-6 expression, thus promoting CSC expansion and increasing their frequency independently of macrophages [52,53]. Likewise, a recent study reported that while the coculture of M2-polarised THP-1 macrophages with MCF-7 cells stimulated the transition to a CSC state (as defined by higher expression of CD44), the addition of recombinant IL-6 did not affect the proportion of these cells [54]. These results reinforce the notion that macrophages are strong modulators of CSC plasticity, while suggesting that the role of IL-6 might be context-dependent. C-X-C motif chemokine ligand 1 (CXCL1) is a chemokine secreted by macrophages whose expression in the tumor stroma is associated with poor prognosis and BC metastasis [55,56]. Interestingly, pharmacological inhibition of TAM-derived CXCL1 suppresses BCSCs [57]. Two complementary studies indicate that other cytokines such as interleukin-1beta (IL-1β) may be involved in the cross-talk between macrophages and BCSCs. For instance, *Trp53^−/−^* cancer cells secrete Wnt ligands that stimulate IL-1β production by macrophages, which in turn activates an inflammatory program that leads to metastasis [58]. Interestingly, IL-1β also triggers Wnt signaling in tumor cells, thus enhancing BCSC features and promoting metastatic colonization to the bone [59]. It is therefore likely that macrophages can promote cancer stemness and metastasis also through the secretion of IL-1β. TAMs may also potentiate BC stemness via cell-cell interactions with CSCs: they express LSECtin, a transmembrane protein that interacts with the butyrofilin family molecule BTN3A3 in tumor cells, enhancing their stemness features both in vitro and in vivo [60]. 

While metastatic dissemination can be an early event in the pathology of BC [61,62], cancer cell dormancy, a process at least partly controlled by TME signals, may prevent the development of clinically detectable metastases for years or decades [63]. Dormant cells have been shown to share some features with CSCs [64]. Interestingly, M2 macrophages can secrete exosomes that maintain the dormancy of CSC-like BC cells, while M1 macrophages trigger the cell cycle via NF-κB [65]. Indeed, mammary epithelial cell NF-κB has also been reported to maintain stemness and to control macrophage recruitment in murine MMTV-ErbbB2 tumors [66].

The effects of TAMs on BCSCs can also be indirect. For instance, hyaluronan synthase 2 (HAS2), an enzyme involved in hyaluronan polymerization in the ECM, is highly expressed by CSCs isolated from human BC cell lines [67]. The binding of hyaluronan to CD44 expressed on TAMs triggers the secretion of platelet-derived growth factor-BB (PDGF-BB), which in turn induces the expression of fibroblast growth factor 7 and 9 (FGF7 and FGF9) in stromal cells, and ultimately stimulates the proliferation of CSCs [67]. Similar to what happens with IL-6, HAS2 overproduction by tumor cells has also been shown to induce BC stemness independently of macrophages through the induction of a transforming growth factor-beta (TGF-β)/Twist-driven EMT program [68]. The interplay between macrophages and CSCs may also change due to microenvironmental conditions. For instance, tumor hypoxia is known to upregulate genes such as IL-6 in macrophages, which, as we have seen, regulates BCSCs [69]. 

Through the secretion of EMT-inducing factors, such as IL-6, TAMs can play an important role in EMT [70], a process which is thought to contribute to the generation of CSCs [23]. For instance, mesenchymal-like BC cells secrete a granulocyte-macrophage colony-stimulating factor (GM-CSF), which stimulates the secretion of C-C motif chemokine ligand 18 (CCL18) in macrophages, promoting EMT and metastasis [71]. Furthermore, experimentally induced EMT triggers the expression of THY1 (CD90) in HMLE cells, and the CD90^high^ population is enriched in stem-like cells [49]. The interactions between macrophages and CSCs mediated through THY1 and EPH receptor A4 (EPHA4) activate NF-κB and sustain cancer stemness [49]. Likewise, the coculture of MCF-7 (luminal A), MDA-MB-231 (TNBC), or T47D (luminal A) cells with M1-polarized THP-1 or peripheral blood mononuclear cell (PBMC)-derived macrophages also promotes the CSC phenotype in a paracrine manner and triggers EMT [72]. The authors found IL-6, together with tumor necrosis factor-alpha (TNF-α) and IL-1β, to be involved in the activation of the EMT program through STAT3/NF-κB, transcriptional upregulation of Lin28B/HMGA2, and downregulation of let-7. Interestingly, this study found M1 macrophages to be stronger inducers of the CSC phenotype than M2 macrophages, and suggests that, as proinflammatory M1 macrophages transdifferentiate into M2 macrophages during tumor progression, the CSC phenotype is maintained—but not expanded [72]. Macrophages have also been involved in the reversibility of the EMT process during the last steps of the metastatic cascade. Indeed, macrophages in the primary and the secondary site are known to possess different traits [73,74]. It was recently shown that in the secondary site, macrophages promote metastatic colonization [75], a process that we previously showed to be led by CSCs [76]. The authors found that whereas in the primary site TAMs mediate EMT through the secretion of TNF-α, in the secondary site metastatic TAMs secrete interleukin-35 (IL-35), which drives MET through the JAK-STAT6-mediated transcription of GATA binding protein 3 (GATA3) [75], a transcription factor known to inhibit metastasis and EMT [77,78].

Myeloid cells can also induce other CSC hallmarks, including chemoresistance. For instance, TAMs and other myeloid cells, such as dendritic cells, secrete milk-fat globule EGF-8 (MFG-E8), which promotes specific CSC chemoresistance in BC cells [79]. In a somewhat reciprocal manner, CSCs isolated from chemoresistant MDA-MB-231-derived cells express interferon regulatory factor 5 (IRF5) and M-CSF, which polarize macrophages to an M2 phenotype and help them acquire tumor-promoting features [80]. Moreover, macrophages can induce the expression of multidrug resistance transporters such as ABCG2 (also known as the breast cancer resistance protein 1, BCRP1) in BCSCs [47], thus potentially enhancing their chemoresistance [81,82]. 

**Figure 1:**
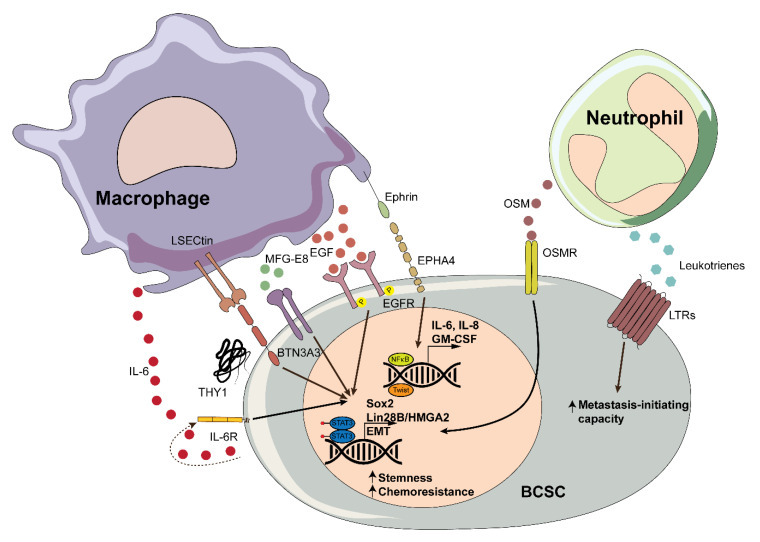
Schematic representation of interactions between myeloid cells and breast cancer stem cells (BCSCs).

In summary, macrophages may promote the CSC phenotype in breast tumors through different mechanisms (Figure 1), including secretion of chemokines, macrophage-BCSC interactions, and involvement of other cell types such as stromal cells. In addition, proangiogenic macrophages contribute to aberrant tumor vasculature, which typically leads to hypoxia, a strong modulator of the BCSC phenotype [83,84,85,86].

#### 3.1.2. Neutrophils

Neutrophils account for most of the granulocytes. They are a phenotypically heterogenous population that plays a role during infection, chronic disease, and cancer. Neutrophils have also been linked to the CSC phenotype in BC. Their prognostic value is evaluated using the neutrophil-to-lymphocyte ratio (NLR): a high NLR is associated with worse disease-free survival and overall survival [87]. Similar to what happens with TAMs, tumor-associated neutrophils (TANs) secrete cytokines that modulate the plasticity of cancer cells. For instance, BC cells induce the production of oncostatin M by TANs [88], which in turn promotes EMT and the BCSC phenotype [89]. TANs also promote EMT and metastasis by secreting the cytokine tissue inhibitor matrix metalloproteinase 1 (TIMP-1) [90]. Similar to the positive feedback loops observed between TAMs and BC cells, the upregulation of THY1 in BC cells undergoing EMT boosts the secretion of TIMP1 by neutrophils. In addition, a different study found that neutrophil-secreted leukotrienes increased the frequency of metastasis-initiating cells, and that the pharmacological or genetic blockade of leukotriene synthesis reduced the metastatic colonization potential in models of mammary gland tumorigenesis [91]. The authors also observed that leukotriene receptors were expressed at higher levels in metastasis-initiating cells.

Overall, the secretion of cytokines and leukotrienes by TANs promotes the BCSC phenotype and the generation of metastasis-initiating cells in BC (Figure 1).

### 3.2. T Cells

T lymphocytes are a main component of the adaptive immune system and are usually classified into cytotoxic CD8^+^ T cells, helper CD4^+^ T cells, regulatory CD4^+^ T cells, and γδ T cells. Cytotoxic CD8^+^ T cells account for most of the tumor infiltrating lymphocytes (TILs), and they typically play a key role in anti-tumor immunity [92]. Indeed, high CD8^+^ infiltration correlates with good prognosis in BC [93,94,95]. However, as cancer progresses, T cells fail to eradicate the tumor, and other components of the immune system such as myeloid cells are reeducated to promote tumor growth. The dual role of the immune system in promoting or suppressing tumor growth is explained by the immunoediting theory, which consists of three phases: elimination, equilibrium, and escape [96]. Although little is known about the particular immunological properties of BCSCs, they may play an important role in tumor immune escape [97]. For instance, BCSCs are known to secrete higher levels of TGF-β [98], a strong suppressor of T cell cytotoxicity [99]. They can also establish immunological tolerance due to the following mechanisms: decreased expression of tumor antigens, downregulation of major histocompatibility complex class-I (MHC I), and inhibition of antigen-presenting cells (APCs) [100]. It is known that cell-cell interactions between BCSCs and CD8^+^ T lymphocytes can impact the killing ability of the latter. One of the main reported immunosuppressive feature of BCSCs consists in programmed death-ligand 1 (PD-L1) expression [101,102], a T-cell inhibitory molecule that binds the cell surface protein PD-1 on effector T cells, causing their exhaustion [103]. Interestingly, human TNBCs expressing high levels of PD-L1 show higher expression of stemness markers, increased capability to form mammospheres, and more tumorigenic potential compared to PD-L1^low^ TNBCs [104]. Additionally, PD-L1^high^ cells were found to be in close contact with infiltrating T lymphocytes, suggesting that they might be able to evade T cell-mediated anti-tumor immunity [104]. Furthermore, Alsuliman et al. reported that PD-L1 expression is induced upon EMT in BC, especially in the claudin-low subtype [105]. Likewise, PD-L1 is important for the expression of the stemness markers OCT4 and Nanog, likely via PI3K/AKT, and concomitantly sustains the expression of the stemness-controlling factor BMI1, independently from the AKT signaling pathway [106]. BCSC activities can in turn be modulated by the T cell compartment. For instance, cognate interactions between killing-deficient CD8^+^ cells and MCF-7 BC cells were found to increase the CD44^high^CD24^low^ stem-like cell population [107]. Moreover, when injected in vivo, the immunologically challenged tumor cells showed a higher capability to form tumors and increased metastatic potential [107]. 

The pro- or anti-tumor functions of CD4^+^ T lymphocytes largely depend on their polarization towards CD4^+^ T helper (Th1, Th2, Th17) or CD4^+^ regulatory T cells (T_reg_). Not much is known about the role of CD4^+^ T cells on BCSCs, but an immunohistochemical analysis of 47 tissue samples from BC patients revealed that CD4^+^ T cells were positively associated to the presence of CD44^+^CD24^-^ BCSCs [108]. 

Immunosuppressive CD4^+^ T_reg_ lymphocytes play a role in inhibiting several immune cell types, including CD8^+^ and some CD4^+^ subsets [109]. Contrary to what happens with CD8^+^ T cells, high T_reg_ infiltration in breast tumors correlates with worse outcomes [110]. While T_regs_ have been recently shown to regulate CSCs in a number of tumor types [111], little is known regarding their role in BC stemness. Nevertheless, T_regs_ have at least been shown to increase the SP and the ALDH^high^ fraction of 4T1 and EO771 BC cells, their capacity to form mammospheres, and their tumorigenic and metastatic potential [112]. Moreover, the overexpression of the stemness factor SOX2 in the tumor cells leads to the recruitment of T_regs_ through CCL1, thus highlighting the bidirectional communication between T_regs_ and BCSCs [112]. However, a recent work showed that the depletion of T_regs_ in early stage lesions in the MMTV-PyMT model led to an unexpected expansion of CD24^−/low^CD44^+^, CD24^+^CD49f^+^, and CD24^+^CD29^high^ stem-like cell subsets and to a progression toward bigger and more invasive tumors [113]. These results were accompanied by a concomitant increase in Th2-derived cytokines and M2 macrophage numbers, suggesting that at earlier stages of tumorigenesis, T_regs_ may play a rather anti-tumorigenic role by modulating the immune landscape and controlling BCSC expansion.

γδ T cells are a small subgroup of T cells that express heterodimeric T-cell receptors (TCR) formed by γ and δ chains. These lymphocytes contribute to the immune response against cancer, and there is an increasing interest in translating their singular properties into novel cancer immunotherapies [114]. In the context of BC, it was shown that HMLER-derived CSC-like cells were resistant to γδ T cell-mediated killing; however, the inhibition of the farnesyl pyrophosphate synthase induced the upregulation of both MHC class I and CD54/ICAM-1, and consequently increased the susceptibility to γδ T and CD8^+^ T cell-mediated lysis [115]. 

**Figure 2:**
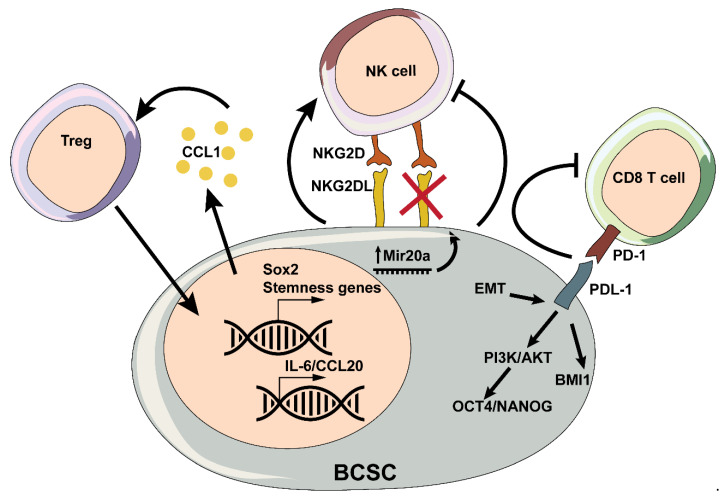
Schematic representation of interactions between T cells, natural killer (NK) cells and BCSCs.

Taken together, the interactions that BCSCs establish with lymphocytes are largely meant to avoid immunosurveillance and therefore to secure BCSC survival. However, the interplay between T cells and BCSCs can also modulate their expansion and self-renewal, as well as their tumor- and metastasis-initiating potential (Figure 2).

### 3.3. Natural Killer Cells

Natural killer (NK) cells are lymphocytes that show both innate and adaptive immune features [116]. NK cell responses depend on interactions between activating or inhibitory receptors with their ligands. Activating receptors typically recognize ligands expressed on transformed cells, while inhibitory receptors bind those expressed on healthy cells [117]. In the presence of an activating signal, NK cells kill the target through two distinct mechanisms: granule exocytosis and death receptor-mediated apoptosis [118]. Due to their cytotoxic role, a high infiltration of NK cells associates with good prognosis in BC [119]. Interestingly, NK cells were shown to preferentially target cells displaying the CSC phenotype, including in MDA-MB-231 BC cells [120]. As a matter of fact, CD44^+^CD24^−^ BCSCs from MCF-7 cells were shown to be more susceptible to the NK-mediated lysis, as they express high levels of UL-16 binding proteins ULBP1 and ULBP2, and MHC class I polypetide-related sequence A (MICA), ligands of the activating NK cell receptor NKG2D [121]. Moreover, Tallerico et al. provided in vitro and in vivo evidence that tumorspheres enriched in CSCs from HER2+ and TNBC cell lines were more susceptible to NK cell killing [122]. 

However, CSCs can escape immunosurveillance through the abrogation of NK cell functions [97]. For instance, it was recently reported that primary human ALDH1^+^ BCSCs become more resistant to NK-cell-mediated cytotoxicity by overexpressing miR20a, which in turn downregulates MICA and MICB, eventually resulting in increased lung metastasis [123]. Interestingly, targeting the miR20a-MICA/B axis with the differentiation agent all-*trans* retinoic acid induced the differentiation of BCSCs and prevented metastasis [123]. 

The capacity of BCSCs to evade NK-mediated killing may also be responsible for the resistance against trastuzumab, a common agent used in the clinic for the treatment of HER2+ tumors. In fact, a study showed that the immunoselection of human BC cell lines with trastuzumab and polyclonal NK cells promoted the preferential survival of the CD44^high^CD24^low^ population, which expresses lower levels of HER2 [124]. Nevertheless, when the selected cells were expanded again, the proportion of CSCs decreased, and the culture was again partially sensitive to NK cytotoxicity—although after repeated cycles of immunoselection the cells acquired a higher tumor initiating-potential and the CD44^high^CD24^low^ population increased [124]. 

Whereas some reports suggest that BCSCs may be more sensitive to NK cells, BCSCs can also avoid NK-cell-mediated killing, which contributes to tumor relapse and metastasis [125] (Figure 2). 

### 3.4. Mesenchymal Stromal Cells and Cancer-Associated Fibroblasts

Mesenchymal stromal cells (MSCs) are multipotent progenitor cells that were first derived from the bone marrow, but ultimately identified in most adult tissues. Besides their potential to differentiate into the osteoblastic, chondrogenic, and adipogenic lineages, they are the precursors of tissue resident fibroblasts. Likewise, tissue resident fibroblasts and MSCs are two main potential cellular origins of CAFs [126]. MSCs have prominent roles in the regulation of tissue inflammation due to their immunomodulatory properties. As with immune cells, MSCs can play dual, opposing roles in BC progression, depending on the context. However, a significant body of evidence indicates that they interact with BCSCs, promoting stemness and tumor progression. Indeed, bone-marrow-derived MSCs can home to breast tumors and fuel growth and metastasis to the lungs, lymph nodes, and bone [127,128,129]. Analogous to what happens with immune cells, MSCs can interact and regulate BCSCs through the secretion of cytokines. Indeed, mixing MSCs and BC cells triggers BC lung metastasis through a CCL5-mediated mechanism involving increased extravasation and motility [130]. Likewise, coculturing experiments show that MSCs promote the BCSC phenotype of SUM159 cells, enhancing their self-renewal capacity [128]. The authors found that this increase was mediated mainly through MSC-secreted CXCL7, and also CXCL5 and CXCL6. CXCL7 was induced, in turn, by cancer cell production of IL-6. As a result of this cytokine cross-talk between BCSCs and MSCs, cancer cells that had previously been in contact with MSCs showed increased capacity to initiate secondary tumors [128]. In line with these results, CCL2 secreted by CAFs potentiated the sphere formation efficiency and self-renewal of BT474 and MDA-MB-361 BC cells through the activation of Notch signaling [131]. In the context of metastasis, MDA-MB-231 derivatives were found to express IL-1 via NF-κB signaling, which promoted the secretion of CXCL9/10 in lung fibroblasts [132]. These two cytokines were found to act on their receptor C-X-C motif chemokine receptor 3 (CXCR3) on metastatic stem cells and boost sphere formation in vitro and promote lung metastasis in vivo [132]. Similarly, a few years ago we reported that the interactions between CSCs and lung fibroblasts are crucial for metastatic colonization [76]. We found that metastasis-initiating cells induce the expression of periostin (POSTN) in lung fibroblasts, which in turn recruits Wnt ligands and presents them to the CSCs, promoting their stemness [76]. 

In Wnt-Met mammary gland tumors, CAFs were found to undergo Hedgehog signaling and to secrete a series of ligands, including ACTIVIN A, which support CSC expansion and self-renewal [133]. Accordingly, in vivo treatment with the Hedgehog inhibitor vismodegib reduced the number of fibroblasts and the ability of tumor cells to form mammospheres. Moreover, Hedgehog activation in CAFs through paracrine signaling with M6 TNBC mammary carcinoma cells induced the secretion of FGF5 and the remodeling of the ECM, which in turn controls BCSC plasticity and promotes chemoresistance [134]. Accordingly, smoothened inhibitors exert strong synergistic effects when combined with docetaxel chemotherapy in preclinical models, a combination therapy that has shown some promising results in an ongoing clinical trial [134]. MSCs and CAFs can also promote the BCSC phenotype through at least two alternative pathways. For instance, when cocultured with MSCs, BC cells expressed mir-199a and mir-214, which fostered CSC propagation and metastasis through the expression of forkhead box P2 (*FOXP2*) [135]. In a different study, autophagic CAFs were found to release high mobility group box-1 (HMGB1), which acted on toll-like receptor 4 (TLR4) to promote the stemness of luminal BC cells [136].

**Figure 3:**
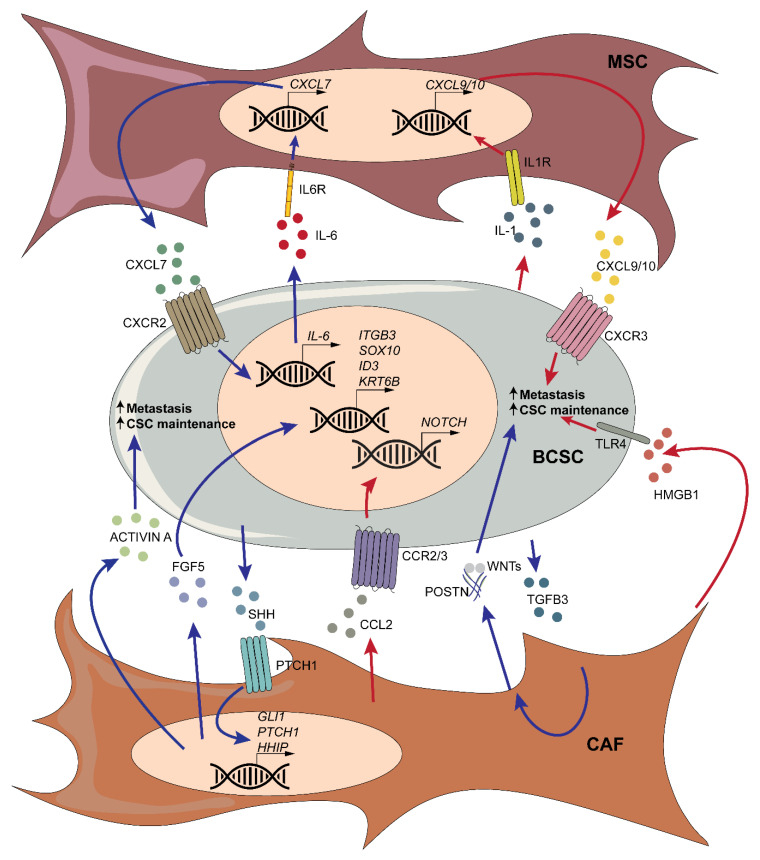
Schematic representation of interactions between mesenchymal stromal cells (MSCs)/cancer-associated fibroblasts (CAFs) and BCSCs.

Briefly, the crosstalk between stromal cells and BCSCs generally promotes stemness, tumor progression, and metastasis (Figure 3). 

### 3.5. Adipose-Derived Mesenchymal Stem Cells and Adipocytes

About 90% of the human breast is made up of adipose tissue, which is composed mainly of fat cells, but also contains macrophages, lymphocytes, endothelial cells, pericytes, fibroblasts, ECM, and adipose precursor cells. The adipose tissue plays important roles during mammary gland development and breast carcinogenesis. Breast adipocytes can be classified into adipose-derived mesenchymal stem cells (ADSCs), preadipocytes, and mature adipocytes. ADSCs share similar properties with MSCs isolated from the bone marrow, and they can contribute to mammary tumorigenesis and metastasis through the activation of a number of pathways [137,138]. For instance, ADSCs secrete adipsin, an adipokine shown to enhance BCSC properties. In particular, adipsin is able to increase the sphere-forming capacity of BC cells and to drive tumor growth [139,140]. Likewise, ADSCs pretreated with the adipokine visfatin and subsequently cocultured with MDA-MB-231 cells enhanced their viability, migration, and tumorsphere formation capacity, as well as tumor growth and metastasis in preclinical models of BC [141]. Interestingly, it was recently shown that ADSCs have the ability to fuse with BC cells and to form cellular hybrids which are enriched in CSCs and have higher tumorigenicity [142]. 

Adipocytes and cancer-associated adipocytes are important players during breast tumorigenesis, as they release a number of factors in the TME, including growth factors, adipokines, hormones, and cytokines that can impact tumor behavior [137,143,144,145,146]. For instance, adipocyte-derived leptin and IL-6 have been shown to activate CSC signaling pathways on adjacent BC cells in a paracrine manner [147,148]. Specifically, upon binding their receptors on target cells, IL-6 and leptin activate the transcription of stemness factors such as OCT4 and Sox2, thus mediating BCSC self-renewal. More recently, adipocyte-derived leptin and its receptor were linked to CSC enrichment and EMT in TNBC [149,150,151]. Interestingly, the leptin-mediated activation of the JAK/STAT3 pathway results in an increase of fatty acid β-oxidation (FAO), which is required for BCSC self-renewal and promotes chemoresistance [152]. As a consequence, targeting the FAO/leptin axis led to decreased BCSC numbers, reduced chemoresistance, and reduced tumor growth in vivo. Similarly, leptin deficiency was found to impair tumor-initiating cells and consequently tumor growth in obese mice [153]. Moreover, interactions between immature adipocytes and BC cells were shown to increase tumor-initiating cell numbers and to drive metastasis formation [154]. Finally, a fourth adipokine, resistin, was found to potentiate the BCSC phenotype and increase tumorsphere formation and EMT [155,156]. 

The impact of the adipose tissue on BCSC biology is exemplified in clinical conditions that involve tissue inflammation, such as obesity. Obesity is characterized by macrophage-driven, low grade, chronic inflammation of the adipose tissue, and it is associated with increased risk of BC and poor prognosis, particularly in post-menopausal women [157,158,159]. We recently showed that, experimentally, obesity triggers EMT and increases the number of metastasis-initiating cells in a mouse model of postmenopausal breast cancer [160]. In addition, Hao and collaborators showed that circulating adipose tissue-derived fatty acid binding protein 4 (FABP4) levels increase during obesity and can activate the axis IL-6/STAT3/ALDH1, thus driving cancer stemness [161].

Given its abundance as well as its capacity to secrete molecules, including growth factors and hormones, the adipose tissue has very relevant functions in mammary gland development, tumorigenesis, and CSC biology. Once they are released in the extracellular space, adipokines modulate the behavior of the neighboring BCSCs, including their capacity to form spheres, initiate tumors, and form metastasis. 

### 3.6. The Extracellular Matrix

The ECM consists of a complex network of proteins that provides structural and functional support to the tissues. It also serves as a reservoir for many secreted growth factors and molecules, thus playing a significant role in regulating cell signaling. Likewise, the ECM is a very important element of the BCSC niche and is a crucial player during BC progression. For instance, matrix stiffening, which depends on the amount of collagen fibers and hyaluronan contained in the tumor mass, promotes malignant transformation [162], alters the vasculature [162], modulates the immune landscape [163], and drives metastasis [164]. Interestingly, ECM stiffness and hypoxia were shown to upregulate the expression of integrin-linked kinase (ILK), which signals through AKT/PI3K and promotes BCSC survival [165]. As a downstream event, ILK upregulates vascular endothelial growth factor A (VEGF-A), thus promoting angiogenesis and the dissemination of breast tumor cells.

As happens with other components of the TME, certain components of the ECM have been proposed to have prognostic value in breast cancer. These include hyaluronan [166,167], asporin [168,169], syndecans [170,171], versican [172,173], collagens [174,175], fibronectin [174,176], transforming growth factor beta-induced (TGFBI) [177], osteopontin (OPN) [178,179], POSTN [180,181], laminins [174,182,183], and tenascin C (TNC) [172,174,184].

#### 3.6.1. Proteoglycans

Proteoglycans (PGs) are one of the major constituents of the ECM. They are heavily glycosylated molecules that can form complexes with other PGs, hyaluronan, and fibrous proteins in the matrix. Interestingly, the G3 domain of versican was found to play a role in modulating BCSC self-renewal by activating EGFR/AKT/GSK-3β signaling and increasing chemoresistance [185]. A second PG, syndecan-1, plays an analogue role in modulating BCSC properties by regulating IL-6/STAT3 [171,186], as well as Notch and EGFR signaling in TNBC, and it has been recently proposed as a novel CSC marker in BC leptomeningeal metastasis [187]. Asporin, a member of the small leucine-rich proteoglycans (SLRP) family, can act as a tumor promoter or a tumor suppressor in BC [168,169,188]. Maris and collaborators found that hormone receptor-positive BC cells induced a strong expression of asporin in breast fibroblasts, whereas TNBC inhibited it through the secretion IL-1β [188]. Asporin was found to inhibit TGF-β-induced EMT, as well as tumor growth and metastasis in TNBC. Accordingly, tumors expressing asporin also contained lower frequencies of ALDH^high^ and CD44^high^/CD24^low^ cells [188]. 

#### 3.6.2. Hyaluronan

The non-proteoglycan polysaccharide hyaluronan (HA) is a very abundant ECM molecule and also a crucial CSC niche factor. It is the only glycosaminoglycan that does not associate with proteins and does not contain any sulfate. The binding of HA to its receptor CD44 triggers the expression of several stem cell markers and promotes drug resistance, cell invasion, and the reorganization of the cytoskeleton in breast cancer cells [189,190]. Moreover, it has been shown that the overproduction of HA drives BC progression through the expansion of the CD44^high^/CD24^low^ cell fraction [68]. ΔNp63, a p53 family member frequently expressed in basal-like BC, is a key regulator of HA family genes, including HA synthase HAS3, hyaluronidase HYAL-1, and CD44. By modulating these pathways, ΔNp63 contributes to creating a microenvironment enriched for HA, which in turn promotes cancer stemness [191]. Additionally, excessive HA production has been shown to activate hypoxia-inducible factor 1 (HIF-1) and consequently BCSC properties through increased hexosamine biosynthetic pathway activity [192].

#### 3.6.3. Collagens

The majority of proteins in the ECM have a fibrotic and non-globular structure, and as a result, they can assemble in more complex networks. Collagens are the most abundant fibrous proteins in the ECM, and they are classified into a number of families according to their structure. Several collagens are involved in modulating the CSC phenotype in BC. For instance, collagen type XIII is a transmembrane protein which is highly expressed in BC and plays a role in enhancing stemness, cell invasion, and resistance to anoikis [193]. Furthermore, a fragment of collagen VI α3 named C5A acts as a ligand for anthrax toxin receptor 1 (ANTXR1), a putative BC stem cell marker [194]. The binding of C5A to ANTXR1 activates Wnt signaling, expands CD24^−^CD44^+^ cells, and boosts metastasis [194]. Collagens are also target genes for stemness-related pathways, and they contribute to tumor initiation and metastasis [134,195]. For instance, the FZD7-WNT5b axis has been shown to modulate the expression of collagen VI, and the knockdown of either FZD7 or COL6A1 in BC cells was reported to decrease invasion and sphere formation [196]. 

#### 3.6.4. Glycoproteins

Glycoproteins can bind to collagens and integrins in order to favor cell migration. The multifunctional protein fibronectin was shown to be the downstream target of the TGF-β/Smad3/COX-2 pathway in TNBC, where it correlates with poor overall survival [197]. In particular, fibronectin regulates BCSC self-renewal through the expansion of ALDH^+^ and CD24^−^CD44^+^ cell populations, revealing its potential use in developing novel targeted therapies for BC [197,198]. In addition, BCSCs can secrete the matricellular protein laminin (LM)-511, which can bind to the integrin α6β1 and subsequently activate tafazzin (TAZ) [199], a component of the Hippo pathway relevant for CSC maintenance [200]. In turn, TAZ modulates the expression of LMα5, the α subunit of LM511, highlighting the existence of a positive loop that sustains BCSC self-renewal and tumor-initiating potential. Conversely, the pretreatment of ER+ LM05-E BC cells with laminin decreases Sox2, Nanog, and Oct-4 through the modulation of the MAPK/ERK pathway, and it also induces tamoxifen resistance mediated by α6 integrin [201]. CSC niches are also enriched for POSTN, a matricellular protein that promotes the initial expansion of BCSCs [76]. POSTN is produced by lung fibroblasts, binds Wnt ligands, and presents them to metastatic stem cells, thus promoting stemness, survival, and growth. The knockdown of POSTN in basal-like BC cell lines impairs mammosphere formation and tumor initiation [202]. As a matter of fact, an intact signaling between POSTN and its receptor αvβ3 integrin can activate the transcription of IL-6 and IL-8, which in turn activate STAT3, crucial for CSC maintenance [202]. POSTN can bind TNC, a glycoprotein that promotes the metastatic outgrowth of disseminated tumor cells in the secondary site. In particular, breast tumor cells that infiltrate the lungs were shown to release high levels of TNC, which drives the expression of musashi RNA binding protein 1 (*MSI1*) and leucine-rich repeat-containing G-protein-coupled receptor 5 (*LGR5*), leading to the activation of the Notch and Wnt pathways, and thus promoting stemness and the outgrowth of pulmonary micrometastases [203]. Moreover, enhanced JNK signaling in BC cells induces the expression of ECM genes, including TNC, that contribute to chemoresistant metastasis [204]. TGFBI, a POSTN paralog, is a 683 aminoacid ECM protein, which contains a secretory signal sequence, an N-terminal cysteine-rich domain (EMI), four fasciclin-1 (FAS1) domains, and an RGD (Arg-Gly-Asp) integrin binding site at the C-terminal end. We have recently identified TGFBI as a novel prognostic factor in BC and a crucial player in modulating interactions between tumor cells and the microenvironment: depletion of TGFBI reduces tumor hypoxia and normalizes the vasculature, thus dramatically decreasing CSC numbers and ultimately impairing metastasis formation [177]. The matricellular phosphoglycoprotein OPN has been shown to trigger the tumorsphere formation of ALDH^high^CD44^+^CD24^−^ BC cells and to boost their metastatic behavior through the interaction with CD44 and RGD-dependent cell surface integrins [205]. Interestingly, OPN was also shown to play a role in controlling the effect of the fibroblast T cell lymphoma invasion and metastasis-inducing factor 1 (Tiam1) in BC, with implications for cancer stemness [206]. In particular, this study showed that Tiam1 expression in fibroblasts modulated the BCSC phenotype in BC cells in vitro and in vivo, and that this effect was dependent on fibroblast OPN. Blocking fibroblast OPN led to a decrease in CD44^+^/CD24^−^/ESA^+^ SUM1315 cell populations, in tumorsphere formation, and prevented lung metastasis [206]. Fibulin-3, also known as EFEMP1, is known to inhibit angiogenesis and was found to be downregulated in sporadic breast carcinomas [207]. Interestingly, EFEMP1 acts as a downstream target of HIF-2α and plays a role in enhancing sphere formation and BCSC self-renewal [208]. Finally, the ECM glycoprotein vitronectin has been identified as another important factor inducing BCSC differentiation and tumor formation upon binding to integrin αvβ3 [209]. 

Altogether, the ECM is a crucial element of the TME that facilitates interactions between different cell types, provides physical and mechanical support for BCSCs, sustains BCSC self-renewal and expansion in the primary and the secondary sites, and promotes tumor progression (Table 1).

## 4. Conclusions

The TME is an essential player driving tumor progression and shaping the evolution and heterogeneity of breast tumors. The complex network of interactions between BCSCs and the TME is crucial to modulate their plasticity and, consequently, the tumor-initiating and metastasis-initiating capabilities of cancer cells. Microenvironmental signals can restrict or promote the acquisition of BCSC traits and modulate the transition of epithelial cancer cells to mesenchymal states, which facilitates some steps of the metastatic cascade. Likewise, in the secondary site, they can trigger the acquisition of epithelial traits and increase the proliferative potential of BCSCs.

The CSC field still suffers from some of the same long-standing problems, such as the lack of universal markers and the recurrently discussed issue of the immunological status of the hosts used for in vivo experiments in CSC research. In vivo studies involving human BC cells are commonly performed in immunocompromised mice (e.g., nude, NOD-SCID, or NSG), which precludes observing the potential interactions between BCSCs and different subsets of immune cells, and usually leads to an overestimation of the CSC content in a tumor. This poses a problem also for the interpretation of the potential pre-clinical trials aiming at disrupting the axis TME-BCSCs. As discussed elsewhere, the use of well-defined mammary gland tumorigenesis mouse models in fully immunocompetent syngeneic settings can help to at least partially overcome this limitation. In parallel, ex vivo organoid culture models such as the air-liquid-interface system [210], which allows for the study of the TME—including the immune compartment—might represent useful tools to explore these interactions and to test therapies targeting the TME. Achieving synergistic effects through combinatorial treatments that target both cancer cells and the TME holds great promise for treating cancer. In this respect, drug repurposing has gathered a considerable momentum over the last few years [211]. For instance, recent studies suggest that aspirin—a non-steroidal anti-inflammatory drug that has become a paradigm of drug repurposing—can act on both BCSCs and certain components of the TME in experimental settings [212,213,214,215,216,217]. Although ongoing clinical trials are still evaluating its potential benefits in BC [218], this example serves as an illustration of the possibilities of such strategies. Additionally, immunotherapy is emerging as a promising tool to manipulate the TME and target BCSCs, directly or indirectly [115,219,220,221,222,223].

Both intrinsic and extrinsic factors determine the BCSC phenotype. Single-cell RNA sequencing studies have revealed specific transcriptional profiles of BCSCs [224,225,226]. In parallel, other studies have focused on unraveling the underlying complexity of the TME. For instance, analyses at the single-cell level have allowed the identification of subgroups of breast CAFs possessing singular transcriptional profiles [227]. Likewise, single-cell RNAseq analyses have uncovered the diversity of the immune TME in breast cancer [228]. Furthermore, the heterogeneity of the TME has been recently explored as a potential prognostic factor in BC, since clustering breast tumors according to their tumor immune microenvironment identifies differences in molecular subtypes, prognosis, proliferation, and EMT features [229]. However, holistic approaches that integrate transcriptional and spatial information may be poised to overcome the drawbacks of single-cell RNA sequencing analyses (i.e., loss of a spatial context and potential underrepresentation of certain cell types due to the need for tissue dissociation) and to provide novel information on extrinsic cues impacting the BCSC phenotype. In this respect, spatial transcriptomics might represent an opportunity to shed more light on how BCSCs interact with the microenvironment in situ, and to study the co-evolution of the TME and BCSCs. Untangling the complexity of the BCSC niche will help design therapies aimed at perturbing the interplay with the microenvironment, restricting plasticity, reducing tumor heterogeneity, and ultimately impairing metastasis.

## Figures and Tables

**Table 1:** Extracellular matrix molecules with BCSC modulatory activities.

ECM Components			Activity	References
Proteoglycans	Versican		Versican modulates BCSC self-renewal by activating EGFR/AKT/GSK-3β signaling.	[185]
	Syndecan-1		Syndecan-1 modulates IL6/STAT3, NOTCH and EGFR signaling in TNBC, and it has been proposed as a novel CSC marker in BC leptomeningeal metastasis.	[171,186,187]
	Small leucin-rich proteoglycans (SLRP)	Asporin	Asporin inhibits TGF-β1-induced EMT, tumor growth, and metastasis; tumors expressing asporin also contain lower frequencies of ALDH^+^ cells and CD44^high^/CD24^low^ cells.	[188]
Non-proteoglycan polysaccharides	Hyaluronan (HA)		HA-CD44 interactions promote the expansion of CD44^high^/CD24^low^, modulate drug resistance, cytoskeleton reorganization, and cell invasion; The p53 family member ΔNp63 contributes to create a microenvironment enriched for HA, which promotes cancer stemness; excessive production of HA activates HIF-1 and BCSC properties.	[68,189,190,191,192]
Fibrous protein	Collagens	Collagen type VI	Binding of collagen VI α3 (C5A) to ANTXR1 activates Wnt signaling, expands CD24^-^CD44^+^ cells, and boosts metastasis; The FZD7-Wnt5b axis modulates the expression of collagen VI, whose knock-out leads to decreased sphere formation ability, tumorigenesis, and metastasis.	[194,195]
		Collagen type XIII	Collagen type XIII is highly expressed in BC and it plays a role in enhancing stemness through β1 integrin, cell invasion, and resistance to anoikis.	[193]
Glycoproteins	Fibronectin		Fibronectin is the downstream target of the TGF-β/Smad3/COX-2 pathway in TNBC; it modulates BCSC self-renewal through the expansion of ALDH^+^ and CD24^-^CD44^+^ cells.	[197,198]
	Laminin	Laminin 511	(LM)-511 binds to α6Bβ1 and activates TAZ, relevant for CSC maintenance; pretreatment of ER+ LM05-E BC cells with laminin decreases Sox-2, Nanog and Oct-4 through modulation of MAPK/ERK pathway, and it induces tamoxifen resistance mediated by α6 integrin.	[199,201]
	Periostin		Lung fibroblast-secreted POSTN recruits Wnt ligands and presents them to metastatic stem cells, thus promoting cell survival and growth; in basal-like BC cell lines, POSTN binds its receptor αvβ3 and activates the transcription of IL6 and IL8, which in turn activates STAT3, crucial for CSC maintenance.	[76,202]
	Tenascin	Tenascin C (TNC)	Breast tumor cells in the lungs release high levels of TNC which drives the expression of MSI1 and LGR5 and promote survival and growth of pulmonary micrometastases; enhanced JNK signaling in BC cells induces the expression of ECM genes including the stem cell niche component TNC that contribute to chemoresistant metastasis.	[203,204]
	TGFBI		Depletion of TGFBI reduces tumor hypoxia and normalizes the vasculature, thus impairing CSC numbers and metastasis formation.	[177]
	Osteopontin		OPN triggers tumorsphere formation and the expansion of ALDH^high^CD44^+^CD24^-^ BC cells and boost their metastatic behaviors through interaction with CD44 and RGD-dependent cell surface integrins; blocking OPN in CAFs leads to a decrease in CD44^+^/CD24^-^/ESA^+^ SUM1315 cell populations and in tumorsphere formation.	[205,206]
	Fibulin	Fibulin-3	Fibulin-3 acts as a downstream target of HIF2α and plays a role in enhancing sphere formation and BCSC self-renewal.	[208]
	Vitronectin		Vitronectin regulates BCSC differentiation and tumor formation upon binding to integrin αvβ3.	[209]

## References

[1] Bray F., Ferlay J., Laversanne M., Brewster D., Mbalawa C.G., Kohler B., Piñeros M., Steliarova-Foucher E., Swaminathan R., Antoni S. (2015). Cancer Incidence in Five Continents: Inclusion criteria, highlights from Volume X and the global status of cancer registration. Int. J. Cancer.

[2] Farmer P., Frenk J., Knaul F.M., Shulman L.N., Alleyne G., Armstrong L., Atun R., Blayney D., Chen L., Feachem R. (2010). Expansion of cancer care and control in countries of low and middle income: A call to action. Lancet.

[3] Harbeck N., Penault-Llorca F., Cortes J., Gnant M., Houssami N., Poortmans P., Ruddy K., Tsang J., Cardoso F. (2019). Breast cancer. Nat. Rev. Dis. Primers.

[4] Wang R., Zhu Y., Liu X., Liao X., He J., Niu L. (2019). The Clinicopathological features and survival outcomes of patients with different metastatic sites in stage IV breast cancer. BMC Cancer.

[5] Rebbeck T.R., Embrace, Friebel T.M., Mitra N., Wan F., Chen S., Andrulis I.L., Apostolou P., Arnold N., Arun B.K. (2016). Inheritance of deleterious mutations at both BRCA1 and BRCA2 in an international sample of 32,295 women. Breast Cancer Res..

[6] Ottini L., Rizzolo P., Silvestri V., Falchetti M. (2011). Inherited and acquired alterations in development of breast cancer. Appl. Clin. Genet..

[7] Zhou J., Chen Q., Zou Y., Chen H., Qi L., Chen Y. (2019). Stem Cells and Cellular Origins of Breast Cancer: Updates in the Rationale, Controversies, and Therapeutic Implications. Front. Oncol..

[8] Batlle E., Clevers H. (2017). Cancer stem cells revisited. Nat. Med..

[9] Brooks M.D., Burness M.L., Wicha M.S. (2015). Therapeutic Implications of Cellular Heterogeneity and Plasticity in Breast Cancer. Cell Stem Cell.

[10] Pece S., Tosoni D., Confalonieri S., Mazzarol G., Vecchi M., Ronzoni S., Bernard L., Viale G., Pelicci P.G., Di Fiore P.P. (2010). Biological and Molecular Heterogeneity of Breast Cancers Correlates with Their Cancer Stem Cell Content. Cell.

[11] Sorlie T., Perou C.M., Tibshirani R., Aas T., Geisler S., Johnsen H., Hastie T., Eisen M.B., van de Rijn M., Jeffrey S.S. (2001). Gene expression patterns of breast carcinomas distinguish tumor subclasses with clinical implications. Proc. Natl. Acad. Sci. USA.

[12] I Herschkowitz J., Simin K., Weigman V.J., Mikaelian I., Usary J., Hu Z., E Rasmussen K., Jones L.P., Assefnia S., Chandrasekharan S. (2007). Identification of conserved gene expression features between murine mammary carcinoma models and human breast tumors. Genome Biol..

[13] Győrffy B., Hatzis C., Sanft T., Hofstatter E., Aktas B., Pusztai L. (2015). Multigene prognostic tests in breast cancer: Past, present, future. Breast Cancer Res..

[14] Ginestier C., Hur M.H., Charafe-Jauffret E., Monville F., Dutcher J., Brown M., Jacquemier J., Viens P., Kleer C.G., Liu S. (2007). ALDH1 Is a Marker of Normal and Malignant Human Mammary Stem Cells and a Predictor of Poor Clinical Outcome. Cell Stem Cell.

[15] Gong Y., Liu Y.-R., Ji P., Hu X., Shao Z.-M. (2017). Impact of molecular subtypes on metastatic breast cancer patients: A SEER population-based study. Sci. Rep..

[16] Kast K., Link T., Friedrich K., Petzold A., Niedostatek A., Schoffer O., Werner C., Klug S.J., Werner A., Gatzweiler A. (2015). Impact of breast cancer subtypes and patterns of metastasis on outcome. Breast Cancer Res. Treat..

[17] Kennecke H., Yerushalmi R., Woods R., Cheang M.C.U., Voduc D., Speers C.H., Nielsen T.O., Gelmon K. (2010). Metastatic Behavior of Breast Cancer Subtypes. J. Clin. Oncol..

[18] Korkaya H., Wicha M.S. (2013). HER2 and Breast Cancer Stem Cells: More than Meets the Eye. Cancer Res..

[19] Hennessy B.T., Gonzalez-Angulo A.-M., Stemke-Hale K., Gilcrease M.Z., Krishnamurthy S., Lee J.-S., Fridlyand J., A Sahin A., Agarwal R., Joy C. (2009). Characterization of a Naturally Occurring Breast Cancer Subset Enriched in Epithelial-to-Mesenchymal Transition and Stem Cell Characteristics. Cancer Res..

[20] Prat A., Parker J.S., Karginova O., Fan C., Livasy C., I Herschkowitz J., He X., Perou C.M. (2010). Phenotypic and molecular characterization of the claudin-low intrinsic subtype of breast cancer. Breast Cancer Res..

[21] Liu S., Cong Y., Wang D., Sun Y., Deng L., Liu Y., Martin-Trevino R., Shang L., McDermott S.P., Landis M.D. (2014). Breast cancer stem cells transition between epithelial and mesenchymal states reflective of their normal counterparts. Stem Cell Rep..

[22] Stankic M., Pavlovic S., Chin Y., Brogi E., Padua D., Norton L., Massague J., Benezra R. (2013). TGF-beta-Id1 signaling opposes Twist1 and promotes metastatic colonization via a mesenchymal-to-epithelial transition. Cell Rep..

[23] Mani S.A., Guo W., Liao M.-J., Eaton E.N., Ayyanan A., Zhou A.Y., Brooks M., Reinhard F., Zhang C.C., Shipitsin M. (2008). The Epithelial-Mesenchymal Transition Generates Cells with Properties of Stem Cells. Cell.

[24] Ye X., Tam W.L., Shibue T., Kaygusuz Y., Reinhardt F., Eaton E.N., Weinberg R.A. (2015). Distinct EMT programs control normal mammary stem cells and tumour-initiating cells. Nat. Cell Biol..

[25] Ocaña O.H., Córcoles R., Fabra Á., Moreno-Bueno G., Acloque H., Vega S., Barrallo-Gimeno A., Cano A., Nieto M.A. (2012). Metastatic Colonization Requires the Repression of the Epithelial-Mesenchymal Transition Inducer Prrx1. Cancer Cell.

[26] Liu X., Li J., Cadilha B.L., Markota A., Voigt C., Huang Z., Lin P.P., Wang D.D., Dai J., Kranz G. (2019). Epithelial-type systemic breast carcinoma cells with a restricted mesenchymal transition are a major source of metastasis. Sci. Adv..

[27] Drasin D.J., Guarnieri A.L., Neelakantan D., Kim J., Cabrera J.H., Wang C.-A., Zaberezhnyy V., Gasparini P., Cascione L., Huebner K. (2015). TWIST1-Induced miR-424 Reversibly Drives Mesenchymal Programming while Inhibiting Tumor Initiation. Cancer Res..

[28] Bierie B., Pierce S.E., Kroeger C., Stover D.G., Pattabiraman D.R., Thiru P., Liu Donaher J., Reinhardt F., Chaffer C.L., Keckesova Z. (2017). Integrin-beta4 identifies cancer stem cell-enriched populations of partially mesenchymal carcinoma cells. Proc. Natl. Acad. Sci. USA.

[29] Fico F., Bousquenaud M., Rüegg C., Santamaria-Martínez A. (2019). Breast Cancer Stem Cells with Tumor- versus Metastasis-Initiating Capacities Are Modulated by TGFBR1 Inhibition. Stem Cell Rep..

[30] Sikandar S.S., Kuo A.H., Kalisky T., Cai S., Zabala M., Hsieh R.W., Lobo N.A., Scheeren F.A., Sim S., Qian D. (2017). Role of epithelial to mesenchymal transition associated genes in mammary gland regeneration and breast tumorigenesis. Nat. Commun..

[31] Santamaria-Martínez A., Huelsken J. (2013). The niche under siege: Novel targets for metastasis therapy. J. Intern. Med..

[32] Peng D., Tanikawa T., Li W., Zhao L., Vatan L., Szeliga W., Wan S., Wei S., Wang Y., Liu Y. (2016). Myeloid-Derived Suppressor Cells Endow Stem-like Qualities to Breast Cancer Cells through IL6/STAT3 and NO/NOTCH Cross-talk Signaling. Cancer Res..

[33] Leek R.D., E Lewis C., Whitehouse R., Greenall M., Clarke J., Harris A.L. (1996). Association of macrophage infiltration with angiogenesis and prognosis in invasive breast carcinoma. Cancer Res..

[34] Al Murri A., Hilmy M., Bell J., Wilson C., McNicol A.-M., Lannigan A., Doughty J.C., McMillan D.C. (2008). The relationship between the systemic inflammatory response, tumour proliferative activity, T-lymphocytic and macrophage infiltration, microvessel density and survival in patients with primary operable breast cancer. Br. J. Cancer.

[35] A Mahmoud S.M., Lee A.H., Paish E.C., Macmillan R.D., O Ellis I., Green A.R. (2011). Tumour-infiltrating macrophages and clinical outcome in breast cancer. J. Clin. Pathol..

[36] Medrek C., Pontén F., Jirström K., Leandersson K. (2012). The presence of tumor associated macrophages in tumor stroma as a prognostic marker for breast cancer patients. BMC Cancer.

[37] Mohammed Z.M.A., Going J.J., Edwards J.W., Elsberger B., Doughty J.C., McMillan D.C. (2012). The relationship between components of tumour inflammatory cell infiltrate and clinicopathological factors and survival in patients with primary operable invasive ductal breast cancer. Br. J. Cancer.

[38] Campbell M.J., Tonlaar N.Y., Garwood E.R., Huo D., Moore D.H., Khramtsov A.I., Au A., Baehner F., Chen Y., Malaka D.O. (2011). Proliferating macrophages associated with high grade, hormone receptor negative breast cancer and poor clinical outcome. Breast Cancer Res. Treat..

[39] Zhang Y., Cheng S., Zhang M., Zhen L., Pang D., Zhang Q., Li Z. (2013). High-Infiltration of Tumor-Associated Macrophages Predicts Unfavorable Clinical Outcome for Node-Negative Breast Cancer. PLoS ONE.

[40] Yuan Z.-Y., Luo R.-Z., Peng R.-J., Wang S.-S., Xue C. (2014). High infiltration of tumor-associated macrophages in triple-negative breast cancer is associated with a higher risk of distant metastasis. OncoTargets Ther..

[41] Tiainen S., Tumelius R., Rilla K., Hämäläinen K., Tammi M., Tammi R., Kosma V.-M., Oikari S., Auvinen P. (2015). High numbers of macrophages, especially M2-like (CD163-positive), correlate with hyaluronan accumulation and poor outcome in breast cancer. Histopathology.

[42] Sousa S., Brion R., Lintunen M., Kronqvist P., Sandholm J., Mönkkönen J., Kellokumpu-Lehtinen P.-L., Lauttia S., Tynninen O., Joensuu H. (2015). Human breast cancer cells educate macrophages toward the M2 activation status. Breast Cancer Res..

[43] Qiu S.-Q., Waaijer S.J.H., Zwager M.C., De Vries E.G.E., Van Der Vegt B., Schröder C.P. (2018). Tumor-associated macrophages in breast cancer: Innocent bystander or important player?. Cancer Treat. Rev..

[44] Timpson P. (2020). Faculty Opinions recommendation of Human Tumor-Associated Macrophage and Monocyte Transcriptional Landscapes Reveal Cancer-Specific Reprogramming, Biomarkers, and Therapeutic Targets. Cancer Cell.

[45] E Gyorki D., Asselin-Labat M.-L., Van Rooijen N., Lindeman G.J., Visvader J.E. (2009). Resident macrophages influence stem cell activity in the mammary gland. Breast Cancer Res..

[46] Chakrabarti R., Celià-Terrassa T., Kumar S., Hang X., Wei Y., Choudhury A., Hwang J., Peng J., Nixon B., Grady J.J. (2018). Notch ligand Dll1 mediates cross-talk between mammary stem cells and the macrophageal niche. Science.

[47] Yang J., Liao D., Chen C., Liu Y., Chuang T.-H., Xiang R., Markowitz D., Reisfeld R.A., Luo Y. (2013). Tumor-Associated Macrophages Regulate Murine Breast Cancer Stem Cells Through a Novel Paracrine EGFR/Stat3/Sox-2 Signaling Pathway. Stem Cells.

[48] Leis O., Eguiara A., Lopez-Arribillaga E., Alberdi M.J., Hernandez-García S., Elorriaga K., Pandiella A., Rezola R., Martín Á.G. (2011). Sox2 expression in breast tumours and activation in breast cancer stem cells. Oncogene.

[49] Lu H., Clauser K.R., Tam W.L., Fröse J., Ye X., Eaton E.N., Reinhardt F., Donnenberg V.S., Bhargava R., Carr S.A. (2014). A breast cancer stem cell niche supported by juxtacrine signalling from monocytes and macrophages. Nat. Cell Biol..

[50] Korkaya H., Liu S., Wicha M.S. (2011). Regulation of cancer stem cells by cytokine networks: Attacking cancer’s inflammatory roots. Clin. Cancer Res..

[51] Marotta L.L., Almendro V., Marusyk A., Shipitsin M., Schemme J., Walker S.R., Bloushtain-Qimron N., Kim J.J., Choudhury S.A., Maruyama R. (2011). The JAK2/STAT3 signaling pathway is required for growth of CD44(+)CD24(−) stem cell-like breast cancer cells in human tumors. J. Clin. Investig..

[52] Weng Y.-S., Tseng H.-Y., Chen Y.-A., Shen P.-C., Al Haq A.T., Chen L.-M., Tung Y.-C., Hsu H.-L. (2019). MCT-1/miR-34a/IL-6/IL-6R signaling axis promotes EMT progression, cancer stemness and M2 macrophage polarization in triple-negative breast cancer. Mol. Cancer.

[53] Kim T., Yang S.-J., Hwang D., Song J., Kim M., Kim S.K., Kang K., Ahn J., Lee D., Kim M.-Y. (2015). A basal-like breast cancer-specific role for SRF–IL6 in YAP-induced cancer stemness. Nat. Commun..

[54] Yang C., Cao M., Liu Y., He Y., Du Y., Zhang G., Gao F. (2019). Inducible formation of leader cells driven by CD44 switching gives rise to collective invasion and metastases in luminal breast carcinomas. Oncogene.

[55] Wang N., Liu W., Zheng Y., Wang S., Yang B., Li M., Song J., Zhang F., Zhang X., Wang Q. (2018). CXCL1 derived from tumor-associated macrophages promotes breast cancer metastasis via activating NF-kappaB/SOX4 signaling. Cell Death Dis..

[56] Zou A., Lambert D., Yeh H., Yasukawa K., Behbod F., Fan F., Cheng N. (2014). Elevated CXCL1 expression in breast cancer stroma predicts poor prognosis and is inversely associated with expression of TGF-beta signaling proteins. BMC Cancer.

[57] Wang S., Liu X., Huang R., Zheng Y., Wang N., Yang B., Situ H., Lin Y., Wang Z. (2019). XIAOPI Formula Inhibits Breast Cancer Stem Cells via Suppressing Tumor-Associated Macrophages/C-X-C Motif Chemokine Ligand 1 Pathway. Front. Pharmacol..

[58] Wellenstein M.D., Coffelt S.B., Duits D.E.M., Van Miltenburg M.H., Slagter M., De Rink I., Henneman L., Kas S.M., Prekovic S., Hau C.-S. (2019). Loss of p53 triggers WNT-dependent systemic inflammation to drive breast cancer metastasis. Nature.

[59] Eyre R., Alferez D.G., Santiago-Gomez A., Spence K., McConnell J.C., Hart C., Simoes B.M., Lefley D., Tulotta C., Storer J. (2019). Microenvironmental IL1beta promotes breast cancer metastatic colonisation in the bone via activation of Wnt signalling. Nat. Commun..

[60] Liu D., Lu Q., Wang X., Wang J., Lu N., Jiang Z., Hao X., Li J., Liu J., Cao P. (2019). LSECtin on tumor-associated macrophages enhances breast cancer stemness via interaction with its receptor BTN3A3. Cell Res..

[61] Hüsemann Y., Geigl J.B., Schubert F., Musiani P., Meyer M., Burghart E., Forni G., Eils R., Fehm T., Riethmüller G. (2008). Systemic Spread Is an Early Step in Breast Cancer. Cancer Cell.

[62] Weng D., Penzner J.H., Song B., Koido S., Calderwood S.K., Gong J. (2012). Metastasis is an early event in mouse mammary carcinomas and is associated with cells bearing stem cell markers. Breast Cancer Res..

[63] Linde N., Fluegen G., Aguirre-Ghiso J.A. (2016). The Relationship between Dormant Cancer Cells and Their Microenvironment. Adv. Cancer Res..

[64] Hen O., Barkan D. (2020). Dormant disseminated tumor cells and cancer stem/progenitor-like cells: Similarities and opportunities. Semin. Cancer Biol..

[65] Walker N.D., Elias M., Guiro K., Bhatia R., Greco S.J., Bryan M., Gergues M., Sandiford O.A., Ponzio N.M., Leibovich S.J. (2019). Exosomes from differentially activated macrophages influence dormancy or resurgence of breast cancer cells within bone marrow stroma. Cell Death Dis..

[66] Liu M., Sakamaki T., Casimiro M.C., Willmarth N.E., Quong A.A., Ju X., Ojeifo J., Jiao X., Yeow W.S., Katiyar S. (2010). The canonical NF-kappaB pathway governs mammary tumorigenesis in transgenic mice and tumor stem cell expansion. Cancer Res..

[67] Okuda H., Kobayashi A., Xia B., Watabe M., Pai S.K., Hirota S., Xing F., Liu W., Pandey P.R., Fukuda K. (2011). Hyaluronan Synthase HAS2 Promotes Tumor Progression in Bone by Stimulating the Interaction of Breast Cancer Stem-Like Cells with Macrophages and Stromal Cells. Cancer Res..

[68] Chanmee T., Ontong P., Mochizuki N., Kongtawelert P., Konno K., Itano N. (2014). Excessive hyaluronan production promotes acquisition of cancer stem cell signatures through the coordinated regulation of Twist and the transforming growth factor beta (TGF-beta)-Snail signaling axis. J. Biol. Chem..

[69] Henze A.-T., Mazzone M. (2016). The impact of hypoxia on tumor-associated macrophages. J. Clin. Investig..

[70] Sullivan N., Sasser A.K., E Axel A., Vesuna F., Raman V., Ramirez N.C., Oberyszyn T.M., Hall B.M. (2009). Interleukin-6 induces an epithelial–mesenchymal transition phenotype in human breast cancer cells. Oncogene.

[71] Su S., Liu Q., Chen J., Chen J., Chen F., He C., Huang D., Wu W., Lin L., Huang W. (2014). A Positive Feedback Loop between Mesenchymal-like Cancer Cells and Macrophages Is Essential to Breast Cancer Metastasis. Cancer Cell.

[72] Guo L., Cheng X., Chen H., Chen C., Xie S., Zhao M., Liu D., Deng Q., Liu Y., Wang X. (2019). Induction of breast cancer stem cells by M1 macrophages through Lin-28B-let-7-HMGA2 axis. Cancer Lett..

[73] Qian B.-Z., Li J., Zhang H., Kitamura T., Zhang J., Campion L.R., Kaiser E.A., Snyder L.A., Pollard J.W. (2011). CCL2 recruits inflammatory monocytes to facilitate breast-tumour metastasis. Nature.

[74] Qian B., Deng Y., Im J.H., Muschel R.J., Zou Y., Li J., Lang R.A., Pollard J.W. (2009). A Distinct Macrophage Population Mediates Metastatic Breast Cancer Cell Extravasation, Establishment and Growth. PLoS ONE.

[75] Lee C.-C., Lin J.-C., Hwang W.-L., Kuo Y.-J., Chen H.-K., Tai S.-K., Lin C., Yang M.-H. (2018). Macrophage-secreted interleukin-35 regulates cancer cell plasticity to facilitate metastatic colonization. Nat. Commun..

[76] Malanchi I., Santamaria-Martínez A., Susanto E., Peng H., Lehr H.-A., Delaloye J.-F., Huelsken J. (2011). Interactions between cancer stem cells and their niche govern metastatic colonization. Nat. Cell Biol..

[77] Yan W., Cao Q.J., Arenas R.B., Bentley B., Shao R. (2010). GATA3 Inhibits Breast Cancer Metastasis through the Reversal of Epithelial-Mesenchymal Transition. J. Biol. Chem..

[78] Kouros-Mehr H., Bechis S.K., Slorach E.M., Littlepage L.E., Egeblad M., Ewald A.J., Pai S.-Y., Ho I.-C., Werb Z. (2008). GATA-3 Links Tumor Differentiation and Dissemination in a Luminal Breast Cancer Model. Cancer Cell.

[79] Jinushi M., Chiba S., Yoshiyama H., Masutomi K., Kinoshita I., Dosaka-Akita H., Yagita H., Takaoka A., Tahara H. (2011). Tumor-associated macrophages regulate tumorigenicity and anticancer drug responses of cancer stem/initiating cells. Proc. Natl. Acad. Sci. USA.

[80] Yamashina T., Baghdadi M., Yoneda A., Kinoshita I., Suzu S., Dosaka-Akita H., Jinushi M. (2014). Cancer Stem-like Cells Derived from Chemoresistant Tumors Have a Unique Capacity to Prime Tumorigenic Myeloid Cells. Cancer Res..

[81] Yuan J.-H., Cheng J.-Q., Jiang L.-Y., Ji W.-D., Guo L.-F., Liu J.-J., Xu X.-Y., He J., Wang X.-M., Zhuang Z.-X. (2008). Breast Cancer Resistance Protein Expression and 5-Fluorouracil Resistance. Biomed. Environ. Sci..

[82] Burger H., A Foekens J., Look M.P., Gelder M.E.M.-V., Klijn J.G.M., Wiemer E.A.C., Stoter G., Nooter K. (2003). RNA expression of breast cancer resistance protein, lung resistance-related protein, multidrug resistance-associated proteins 1 and 2, and multidrug resistance gene 1 in breast cancer: Correlation with chemotherapeutic response. Clin. Cancer Res..

[83] Conley S.J., Gheordunescu E., Kakarala P., Newman B., Korkaya H., Heath A.N., Clouthier S.G., Wicha M.S. (2012). Antiangiogenic agents increase breast cancer stem cells via the generation of tumor hypoxia. Proc. Natl. Acad. Sci. USA.

[84] De Palma M., Biziato D., Petrova T.V. (2017). Microenvironmental regulation of tumour angiogenesis. Nat. Rev. Cancer.

[85] Kim H., Lin Q., Glazer P.M., Yun Z. (2018). The hypoxic tumor microenvironment in vivo selects the cancer stem cell fate of breast cancer cells. Breast Cancer Res..

[86] Schwab L.P., Peacock D.L., Majumdar D., Ingels J.F., Jensen L.C., Smith K.D., Cushing R.C., Seagroves T.N. (2012). Hypoxia-inducible factor 1α promotes primary tumor growth and tumor-initiating cell activity in breast cancer. Breast Cancer Res..

[87] Ethier J.-L., Desautels D., Templeton A., Shah P.S., Amir E. (2017). Prognostic role of neutrophil-to-lymphocyte ratio in breast cancer: A systematic review and meta-analysis. Breast Cancer Res..

[88] Queen M.M., Ryan R.E., Holzer R.G., Keller-Peck C.R., Jorcyk C.L. (2005). Breast Cancer Cells Stimulate Neutrophils to Produce Oncostatin M: Potential Implications for Tumor Progression. Cancer Res..

[89] Junk D.J., Bryson B.L., Smigiel J.M., Parameswaran N., A Bartel C., Jackson M.W. (2017). Oncostatin M promotes cancer cell plasticity through cooperative STAT3-SMAD3 signaling. Oncogene.

[90] Wang Y., Chen J.-N., Yang L., Li J., Wu W., Huang M., Lin L., Su S. (2018). Tumor-Contacted Neutrophils Promote Metastasis by a CD90-TIMP-1 Juxtacrine–Paracrine Loop. Clin. Cancer Res..

[91] Wculek S.K., Malanchi I. (2015). Neutrophils support lung colonization of metastasis-initiating breast cancer cells. Nat. Cell Biol..

[92] Zhang N., Bevan M.J. (2011). CD8+ T Cells: Foot Soldiers of the Immune System. Immunity.

[93] Ali H.R., Provenzano E., Dawson S.-J., Blows F.M., Liu B., Shah M., Earl H.M., Poole C.J., Hiller L., Dunn J.A. (2014). Association between CD8+ T-cell infiltration and breast cancer survival in 12 439 patients. Ann. Oncol..

[94] Mahmoud S.M., Paish E.C., Powe D.G., Macmillan R.D., Grainge M.J., Lee A.H.S., Ellis I.O., Green A.R. (2011). Tumor-Infiltrating CD8+ Lymphocytes Predict Clinical Outcome in Breast Cancer. J. Clin. Oncol..

[95] Adams S., Gray R.J., DeMaria S., Goldstein L., Perez E.A., Shulman L.N., Martino S., Wang M., Jones V.E., Saphner T.J. (2014). Prognostic Value of Tumor-Infiltrating Lymphocytes in Triple-Negative Breast Cancers From Two Phase III Randomized Adjuvant Breast Cancer Trials: ECOG 2197 and ECOG 1199. J. Clin. Oncol..

[96] Gil Del Alcazar C.R., Alečković M., Polyak K. (2020). Immune Escape during Breast Tumor Progression. Cancer Immunol. Res..

[97] Bruttel V.S., Wischhusen J., Wischhusen J. (2014). Cancer Stem Cell Immunology: Key to Understanding Tumorigenesis and Tumor Immune Escape?. Front. Immunol..

[98] Shipitsin M., Campbell L.L., Argani P., Weremowicz S., Bloushtain-Qimron N., Yao J., Nikolskaya T., Serebryiskaya T., Beroukhim R., Hu M. (2007). Molecular Definition of Breast Tumor Heterogeneity. Cancer Cell.

[99] Thomas D.A., Massague J. (2005). TGF-beta directly targets cytotoxic T cell functions during tumor evasion of immune surveillance. Cancer Cell.

[100] Codony-Servat J., Rosell R. (2015). Cancer stem cells and immunoresistance: Clinical implications and solutions. Transl. Lung Cancer Res..

[101] Boyle S.T., Kochetkova M. (2014). Breast Cancer Stem Cells and the Immune System: Promotion, Evasion and Therapy. J. Mammary Gland. Biol. Neoplasia.

[102] Sumransub N., Jirapongwattana N., Jamjuntra P., Thongchot S., Chieochansin T., Yenchitsomanus P.T., Thuwajit P., Warnnissorn M., O-Charoenra P., Thuwajit C. (2020). Breast cancer stem cell RNA-pulsed dendritic cells enhance tumor cell killing by effector T cells. Oncol. Lett..

[103] Wu Y., Chen C., Xu Z.P., Gu W. (2017). Increased PD-L1 expression in breast and colon cancer stem cells. Clin. Exp. Pharmacol. Physiol..

[104] Castagnoli L., Cancila V., Cordoba-Romero S.L., Faraci S., Talarico G., Belmonte B., Iorio M.V., Milani M., Volpari T., Chiodoni C. (2019). WNT signaling modulates PD-L1 expression in the stem cell compartment of triple-negative breast cancer. Oncogene.

[105] Alsuliman A., Colak D., Al-Harazi O., Fitwi H., Tulbah A., Al-Tweigeri T., Al-Alwan M., Ghebeh H. (2015). Bidirectional crosstalk between PD-L1 expression and epithelial to mesenchymal transition: Significance in claudin-low breast cancer cells. Mol. Cancer.

[106] Almozyan S., Colak D., Mansour F., Alaiya A., Al-Harazi O., Qattan A., Al-Mohanna F., Al-Alwan M., Ghebeh H. (2017). PD-L1 promotes OCT4 and Nanog expression in breast cancer stem cells by sustaining PI3K/AKT pathway activation. Int. J. Cancer.

[107] Stein R.G., Ebert S., Schlahsa L., Scholz C.J., Braun M., Hauck P., Horn E., Monoranu C.-M., Thiemann V.J., Wustrow M.P. (2019). Cognate Nonlytic Interactions between CD8+ T Cells and Breast Cancer Cells Induce Cancer Stem Cell–like Properties. Cancer Res..

[108] Jeong Y.J., Oh H.K., Park S.H., Bong J.G. (2017). Association between inflammation and cancer stem cell phenotype in breast cancer. Oncol. Lett..

[109] Togashi Y., Shitara K., Nishikawa H. (2019). Regulatory T cells in cancer immunosuppression — implications for anticancer therapy. Nat. Rev. Clin. Oncol..

[110] Shou J., Zhang Z., Lai Y., Chen Z., Huang J. (2016). Worse outcome in breast cancer with higher tumor-infiltrating FOXP3+ Tregs: A systematic review and meta-analysis. BMC Cancer.

[111] Yu X., Li H., Ren X. (2012). Interaction between regulatory T cells and cancer stem cells. Int. J. Cancer.

[112] Xu Y., Dong X., Qi P., Ye Y., Shen W., Leng L., Wang L., Li X., Luo X., Chen Y. (2017). Sox2 Communicates with Tregs Through CCL1 to Promote the Stemness Property of Breast Cancer Cells. Stem Cells.

[113] Martinez L.M., Robila V., Clark N.M., Du W., Idowu M.O., Rutkowski M.R., Bos P.D. (2019). Regulatory T Cells Control the Switch From in situ to Invasive Breast Cancer. Front. Immunol..

[114] Zhao Y., Niu C., Cui J. (2018). Gamma-delta (gammadelta) T cells: Friend or foe in cancer development?. J. Transl. Med..

[115] Chen H.C., Joalland N., Bridgeman J.S., Alchami F.S., Jarry U., Khan M.W.A., Piggott L., Shanneik Y., Li J., Herold M.J. (2017). Synergistic targeting of breast cancer stem-like cells by human gammadelta T cells and CD8(+) T cells. Immunol. Cell Biol..

[116] Raulet D.H. (2004). Interplay of natural killer cells and their receptors with the adaptive immune response. Nat. Immunol..

[117] Pegram H.J., Andrews D.M., Smyth M.J., Darcy P.K., Kershaw M.H. (2011). Activating and inhibitory receptors of natural killer cells. Immunol. Cell Biol..

[118] Höglund P., Klein E. (2006). Natural killer cells in cancer. Semin. Cancer Biol..

[119] Habif G., Crinier A., André P., Vivier E., Narni-Mancinelli É. (2019). Targeting natural killer cells in solid tumors. Cell. Mol. Immunol..

[120] Ames E., Canter R.J., Grossenbacher S.K., Mac S., Chen M., Smith R.C., Hagino T., Perez-Cunningham J., Sckisel G.D., Urayama S. (2015). NK Cells Preferentially Target Tumor Cells with a Cancer Stem Cell Phenotype. J. Immunol..

[121] Yin T., Wang G., He S., Liu Q., Sun J., Wang Y. (2016). Human cancer cells with stem cell-like phenotype exhibit enhanced sensitivity to the cytotoxicity of IL-2 and IL-15 activated natural killer cells. Cell. Immunol..

[122] Tallerico R., Conti L., Lanzardo S., Sottile R., Garofalo C., Wagner A.K., Johansson M.H., Cristiani C.M., Kärre K., Carbone E. (2017). NK cells control breast cancer and related cancer stem cell hematological spread. Oncoimmunology.

[123] Wang B., Wang Q., Wang Z., Jiang J., Yu S.-C., Ping Y.-F., Yang J., Xu S.-L., Ye X.-Z., Xu C. (2014). Metastatic Consequences of Immune Escape from NK Cell Cytotoxicity by Human Breast Cancer Stem Cells. Cancer Res..

[124] Reim F., Dombrowski Y., Ritter C., Buttmann M., Häusler S., Ossadnik M., Krockenberger M., Beier D., Beier C.P., Dietl J. (2009). Immunoselection of Breast and Ovarian Cancer Cells with Trastuzumab and Natural Killer Cells: Selective Escape of CD44high/CD24low/HER2low Breast Cancer Stem Cells. Cancer Res..

[125] Sultan M., Coyle K.M., Vidovic D., Thomas M.L., Gujar S., Marcato P. (2016). Hide-and-seek: The interplay between cancer stem cells and the immune system. Carcinogenesis.

[126] Shiga K., Hara M., Nagasaki T., Sato T., Takahashi H., Takeyama H. (2015). Cancer-Associated Fibroblasts: Their Characteristics and Their Roles in Tumor Growth. Cancers.

[127] Goldstein R.H., Reagan M.R., Anderson K., Kaplan D.L., Rosenblatt M. (2010). Human Bone Marrow-Derived MSCs Can Home to Orthotopic Breast Cancer Tumors and Promote Bone Metastasis. Cancer Res..

[128] Liu S., Ginestier C., Ou S.J., Clouthier S.G., Patel S.H., Monville F., Korkaya H., Heath A., Dutcher J., Kleer C.G. (2011). Breast Cancer Stem Cells Are Regulated by Mesenchymal Stem Cells through Cytokine Networks. Cancer Res..

[129] Chaturvedi P., Gilkes D.M., Wong C.C.-L., Kshitiz, Luo W., Zhang H., Wei H., Takano N., Schito L., Levchenko A. (2012). Hypoxia-inducible factor–dependent breast cancer–mesenchymal stem cell bidirectional signaling promotes metastasis. J. Clin. Investig..

[130] Karnoub A.E., Dash A.B., Vo A.P., Sullivan A., Brooks M.W., Bell G.W., Richardson A.L., Polyak K., Tubo R., Weinberg R.A. (2007). Mesenchymal stem cells within tumour stroma promote breast cancer metastasis. Nat. Cell Biol..

[131] Tsuyada A., Chow A., Wu J., Somlo G., Chu P., Loera S., Luu T., Li A.X., Wu X., Ye W. (2012). CCL2 Mediates Cross-talk between Cancer Cells and Stromal Fibroblasts That Regulates Breast Cancer Stem Cells. Cancer Res..

[132] Pein M., Insua-Rodríguez J., Hongu T., Riedel A., Meier J., Wiedmann L., Decker K., Essers M.A.G., Vogt P.H., Spaich S. (2020). Metastasis-initiating cells induce and exploit a fibroblast niche to fuel malignant colonization of the lungs. Nat. Commun..

[133] Valenti G., Quinn H.M., Heynen G.J., Lan L., Holland J.D., Vogel R., Wulf-Goldenberg A., Birchmeier W. (2017). Cancer Stem Cells Regulate Cancer-Associated Fibroblasts via Activation of Hedgehog Signaling in Mammary Gland Tumors. Cancer Res..

[134] Cazet A.S., Hui M.N., Elsworth B.L., Wu S.Z., Roden D., Chan C.L., Skhinas J.N., Collot R., Yang J., Harvey K. (2018). Targeting stromal remodeling and cancer stem cell plasticity overcomes chemoresistance in triple negative breast cancer. Nat. Commun..

[135] Cuiffo B.G., Campagne A., Bell G.W., Lembo A., Orso F., Lien E.C., Bhasin M.K., Raimo M., Hanson S.E., Marusyk A. (2014). MSC-Regulated MicroRNAs Converge on the Transcription Factor FOXP2 and Promote Breast Cancer Metastasis. Cell Stem Cell.

[136] Zhao X.-L., Lin Y., Jiang J., Tang Z., Yang S., Lu L., Liang Y., Liu X., Tan J., Hu X.-G. (2017). High-mobility group box 1 released by autophagic cancer-associated fibroblasts maintains the stemness of luminal breast cancer cells. J. Pathol..

[137] Muehlberg F., Song Y.-H., Krohn A., Pinilla S.P., Droll L.H., Leng X., Seidensticker M., Ricke J., Altman A.M., Devarajan E. (2009). Tissue-resident stem cells promote breast cancer growth and metastasis. Carcinogenesis.

[138] Koellensperger E., Bonnert L.-C., Zörnig I., Marmé F., Sandmann S., Germann G., Gramley F., Leimer U. (2017). The impact of human adipose tissue-derived stem cells on breast cancer cells: Implications for cell-assisted lipotransfers in breast reconstruction. Stem Cell Res. Ther..

[139] Chen Y., He Y., Wang X., Lu F., Gao J. (2019). Adipose-derived mesenchymal stem cells exhibit tumor tropism and promote tumorsphere formation of breast cancer cells. Oncol. Rep..

[140] Goto H., Shimono Y., Funakoshi Y., Imamura Y., Toyoda M., Kiyota N., Kono S., Takao S., Mukohara T., Minami H. (2018). Adipose-derived stem cells enhance human breast cancer growth and cancer stem cell-like properties through adipsin. Oncogene.

[141] Huang J.-Y., Wang Y.-Y., Lo S.J., Tseng L.-M., Chen D.-R., Wu Y.-C., Hou M.-F., Yuan S.-S.F. (2019). Visfatin Mediates Malignant Behaviors through Adipose-Derived Stem Cells Intermediary in Breast Cancer. Cancers.

[142] Chan Y.W., So C., Yau K.L., Chiu K.C., Wang X., Chan F.L., Tsang S.-Y. (2020). Adipose-derived stem cells and cancer cells fuse to generate cancer stem cell-like cells with increased tumorigenicity. J. Cell. Physiol..

[143] Zhao Y., Zhang X., Zhao H., Wang J., Zhang Q. (2017). CXCL5 secreted from adipose tissue-derived stem cells promotes cancer cell proliferation. Oncol. Lett..

[144] Dirat B., Bochet L., Dabek M., Daviaud D., Dauvillier S., Majed B., Wang Y.Y., Meulle A., Salles B., Le Gonidec S. (2011). Cancer-Associated Adipocytes Exhibit an Activated Phenotype and Contribute to Breast Cancer Invasion. Cancer Res..

[145] Wu Q., Li B., Li Z., Li J., Sun S., Sun S. (2019). Cancer-associated adipocytes: Key players in breast cancer progression. J. Hematol. Oncol..

[146] Chu D.-T., Dinh T.C., Tien N.L.B., Tran D.-K., Nguyen T.-T., Van Thanh V., Quang T.L., Bui L.M., Pham V.H., Ngoc V.T.N. (2019). The Effects of Adipocytes on the Regulation of Breast Cancer in the Tumor Microenvironment: An Update. Cells.

[147] Wolfson B., Eades G., Zhou Q. (2015). Adipocyte activation of cancer stem cell signaling in breast cancer. World J. Biol. Chem..

[148] Mishra A.K., Parish C.R., Wong M.-L., Licinio J., Blackburn A.C. (2017). Leptin signals via TGFB1 to promote metastatic potential and stemness in breast cancer. PLoS ONE.

[149] Zheng Q., Banaszak L., Fracci S., Basali D., Dunlap S.M., Hursting S.D., Rich J.N., Hjlemeland A.B., Vasanji A., Berger N.A. (2013). Leptin receptor maintains cancer stem-like properties in triple negative breast cancer cells. Endocr.-Relat. Cancer.

[150] Thiagarajan P.S., Zheng Q., Bhagrath M., Mulkearns-Hubert E., Myers M.G., Lathia J.D., Reizes O. (2017). STAT3 activation by leptin receptor is essential for TNBC stem cell maintenance. Endocr.-Relat. Cancer.

[151] Bowers L.W., Rossi E.L., McDonell S.B., Doerstling S.S., Khatib S.A., Lineberger C.G., Albright J.E., Tang X., Degraffenried L.A., Hursting S.D. (2018). Leptin Signaling Mediates Obesity-Associated CSC Enrichment and EMT in Preclinical TNBC Models. Mol. Cancer Res..

[152] Wang T., Fahrmann J.F., Lee H., Li Y.J., Tripathi S.C., Yue C., Zhang C., Lifshitz V., Song J., Yuan Y. (2018). JAK/STAT3-Regulated Fatty Acid beta-Oxidation Is Critical for Breast Cancer Stem Cell Self-Renewal and Chemoresistance. Cell Metab..

[153] Zheng Q., Dunlap S.M., Zhu J., Downs-Kelly E., Rich J.N., Hursting S.D., Berger N.A., Reizes O. (2011). Leptin deficiency suppresses MMTV-Wnt-1 mammary tumor growth in obese mice and abrogates tumor initiating cell survival. Endocr.-Relat. Cancer.

[154] Picon-Ruiz M., Pan C., Drews-Elger K., Jang K., Besser A.H., Zhao D., Morata-Tarifa C., Kim M., Ince T.A., Azzam D.J. (2016). Interactions between Adipocytes and Breast Cancer Cells Stimulate Cytokine Production and Drive Src/Sox2/miR-302b–Mediated Malignant Progression. Cancer Res..

[155] Wang C.-H., Wang P.-J., Hsieh Y.-C., Lo S., Lee Y.-C., Chen Y.-C., Tsai C.-H., Chiu W.-C., Hu S.C.-S., Lu C.-W. (2018). Resistin facilitates breast cancer progression via TLR4-mediated induction of mesenchymal phenotypes and stemness properties. Oncogene.

[156] Avtanski D., Garcia A., Caraballo B., Thangeswaran P., Marin S., Bianco J., Lavi A., Poretsky L. (2019). Resistin induces breast cancer cells epithelial to mesenchymal transition (EMT) and stemness through both adenylyl cyclase-associated protein 1 (CAP1)-dependent and CAP1-independent mechanisms. Cytokine.

[157] Weisberg S.P., McCann D., Desai M., Rosenbaum M., Leibel R.L., Ferrante A.W. (2003). Obesity is associated with macrophage accumulation in adipose tissue. J. Clin. Investig..

[158] Lumeng C.N., Bodzin J.L., Saltiel A.R. (2007). Obesity induces a phenotypic switch in adipose tissue macrophage polarization. J. Clin. Investig..

[159] Jiralerspong S., Goodwin P.J. (2016). Obesity and Breast Cancer Prognosis: Evidence, Challenges, and Opportunities. J. Clin. Oncol..

[160] Bousquenaud M., Fico F., Solinas G., Rüegg C., Santamaria-Martínez A. (2018). Obesity promotes the expansion of metastasis-initiating cells in breast cancer. Breast Cancer Res..

[161] Hao J., Zhang Y., Yan X., Yan F., Sun Y., Zeng J., Waigel S., Yin Y., Fraig M.M., Egilmez N.K. (2018). Circulating Adipose Fatty Acid Binding Protein Is a New Link Underlying Obesity-Associated Breast/Mammary Tumor Development. Cell Metab..

[162] Ondeck M.G., Kumar A., Placone J.K., Plunkett C.M., Matte B.F., Wong K.C., Fattet L., Yang J., Engler A.J. (2019). Dynamically stiffened matrix promotes malignant transformation of mammary epithelial cells via collective mechanical signaling. Proc. Natl. Acad. Sci. USA.

[163] García-Mendoza M.G., Inman D.R., Ponik S.M., Jeffery J.J., Sheerar D.S., Van Doorn R.R., Keely P.J. (2016). Neutrophils drive accelerated tumor progression in the collagen-dense mammary tumor microenvironment. Breast Cancer Res..

[164] Wei S.C., Fattet L., Tsai J.H., Guo Y., Pai V.H., Majeski H.E., Chen A.C., Sah R.L., Taylor S.S., Engler A.J. (2015). Matrix stiffness drives epithelial–mesenchymal transition and tumour metastasis through a TWIST1–G3BP2 mechanotransduction pathway. Nat. Cell Biol..

[165] Pang M.-F., Siedlik M.J., Han S., Stallings-Mann M., Radisky D.C., Nelson C.M. (2016). Tissue Stiffness and Hypoxia Modulate the Integrin-Linked Kinase ILK to Control Breast Cancer Stem-like Cells. Cancer Res..

[166] Auvinen P., Tammi R., Parkkinen J., Tammi M., Ågren U., Johansson R., Hirvikoski P., Eskelinen M., Kosma V.-M. (2000). Hyaluronan in Peritumoral Stroma and Malignant Cells Associates with Breast Cancer Spreading and Predicts Survival. Am. J. Pathol..

[167] Corte M.D., González L., Lamelas M.L., Álvarez A., Junquera S., Allende M.T., García-Muñiz J.L., Argüelles J., Vizoso F., Argüelles J. (2006). Expression and Clinical Signification of Cytosolic Hyaluronan Levels in Invasive Breast Cancer. Breast Cancer Res. Treat..

[168] Castellana B., Escuin D., Peiró G., Garcia-Valdecasas B., Vázquez T., Pons C., Pérez-Olabarria M., Barnadas A., Lerma E. (2012). ASPN and GJB2 Are Implicated in the Mechanisms of Invasion of Ductal Breast Carcinomas. J. Cancer.

[169] Simkova D., Kharaishvili G., Korinkova G., Oždian T., Kleplová T.S., Soukup T., Krupka M., Galandáková A., Dzubak P., Janikova M. (2016). The dual role of asporin in breast cancer progression. Oncotarget.

[170] Barbareschi M., Maisonneuve P., Aldovini D., Cangi M.G., Pecciarini L., Angelo Mauri F., Veronese S., Caffo O., Lucenti A., Palma P.D. (2003). High syndecan-1 expression in breast carcinoma is related to an aggressive phenotype and to poorer prognosis. Cancer.

[171] Ibrahim S.A., Gadalla R., El-Ghonaimy E.A., Samir O., Mohamed H.T., Hassan H., Greve B., El-Shinawi M., Mohamed M.M., Götte M. (2017). Syndecan-1 is a novel molecular marker for triple negative inflammatory breast cancer and modulates the cancer stem cell phenotype via the IL-6/STAT3, Notch and EGFR signaling pathways. Mol. Cancer.

[172] Suwiwat S. (2004). Expression of Extracellular Matrix Components Versican, Chondroitin Sulfate, Tenascin, and Hyaluronan, and Their Association with Disease Outcome in Node-Negative Breast Cancer. Clin. Cancer Res..

[173] Ricciardelli C., Brooks J.H., Suwiwat S., Sakko A.J., Mayne K., Raymond W.A., Seshadri R., LeBaron R.G., Horsfall D.J. (2002). Regulation of stromal versican expression by breast cancer cells and importance to relapse-free survival in patients with node-negative primary breast cancer. Clin. Cancer Res..

[174] Ioachim E.E., Charchanti A., Briasoulis E., Karavasilis V., Tsanou H., Arvanitis D., Agnantis N., Pavlidis N. (2002). Immunohistochemical expression of extracellular matrix components tenascin, fibronectin, collagen type IV and laminin in breast cancer: Their prognostic value and role in tumour invasion and progression. Eur. J. Cancer.

[175] Van’t Veer L.J., Dai H., van de Vijver M.J., He Y.D., Hart A.A., Mao M., Peterse H.L., van der Kooy K., Marton M.J., Witteveen A.T. (2002). Gene expression profiling predicts clinical outcome of breast cancer. Nature.

[176] Fernandez-Garcia B., Eiró N., Marín L., González-Reyes S., González L.O., Lamelas M.L., Vizoso F.J. (2013). Expression and prognostic significance of fibronectin and matrix metalloproteases in breast cancer metastasis. Histopathology.

[177] Fico F., Santamaria-Martínez A. (2020). TGFBI modulates tumour hypoxia and promotes breast cancer metastasis. Mol. Oncol..

[178] Anborgh P.H., Caria L.B., Chambers A.F., Tuck A.B., Stitt L.W., Brackstone M. (2015). Role of plasma osteopontin as a biomarker in locally advanced breast cancer. Am. J. Transl. Res..

[179] Bramwell V.H. (2006). Serial Plasma Osteopontin Levels Have Prognostic Value in Metastatic Breast Cancer. Clin. Cancer Res..

[180] Xu D., Xu H., Ren Y., Liu C., Wang X., Zhang H., Lu P. (2012). Cancer Stem Cell-Related Gene Periostin: A Novel Prognostic Marker for Breast Cancer. PLoS ONE.

[181] Li C., Xu J., Wang Q., Geng S., Yan Z., You J., Li Z., Zou X. (2018). Prognostic value of periostin in early-stage breast cancer treated with conserving surgery and radiotherapy. Oncol. Lett..

[182] Carpenter P.M., Ziogas A., Markham E.M., Cantillep A.S., Yan R., Anton-Culver H. (2018). Laminin 332 expression and prognosis in breast cancer. Hum. Pathol..

[183] Chia J., Kusuma N., Anderson R.L., Parker B.S., Bidwell B., Zamurs L., Nice E., Pouliot N. (2007). Evidence for a Role of Tumor-Derived Laminin-511 in the Metastatic Progression of Breast Cancer. Am. J. Pathol..

[184] Wawrzyniak D., Grabowska M., Głodowicz P., Kuczyński K., Kuczyńska B., Fedoruk-Wyszomirska A., Rolle K. (2020). Down-regulation of tenascin-C inhibits breast cancer cells development by cell growth, migration, and adhesion impairment. PLoS ONE.

[185] Du W.W., Fang L., Yang X., Sheng W., Yang B.L., Seth A., Zhang Y., Yang B.B., Yee A.J. (2013). The Role of Versican in Modulating Breast Cancer Cell Self-renewal. Mol. Cancer Res..

[186] Ibrahim S.A., Hassan H., Vilardo L., Kumar S.K., Kumar A.V., Kelsch R., Schneider C., Kiesel L., Eich H.T., Zucchi I. (2013). Syndecan-1 (CD138) Modulates Triple-Negative Breast Cancer Stem Cell Properties via Regulation of LRP-6 and IL-6-Mediated STAT3 Signaling. PLoS ONE.

[187] Cordone I., Masi S., Summa V., Carosi M., Vidiri A., Fabi A., Pasquale A., Conti L., Rosito I., Carapella C. (2017). Overexpression of syndecan-1, MUC-1, and putative stem cell markers in breast cancer leptomeningeal metastasis: A cerebrospinal fluid flow cytometry study. Breast Cancer Res..

[188] Maris P., Blomme A., Palacios A.P., Costanza B., Bellahcene A., Bianchi E., Gofflot S., Drion P., Trombino G.E., Di Valentin E. (2015). Asporin Is a Fibroblast-Derived TGF-beta1 Inhibitor and a Tumor Suppressor Associated with Good Prognosis in Breast Cancer. PLoS Med..

[189] Bourguignon L.Y.W., Peyrollier K., Xia W., Gilad E. (2008). Hyaluronan-CD44 Interaction Activates Stem Cell Marker Nanog, Stat-3-mediated MDR1 Gene Expression, and Ankyrin-regulated Multidrug Efflux in Breast and Ovarian Tumor Cells. J. Biol. Chem..

[190] Bourguignon L.Y.W., Wong G., Earle C., Krueger K., Spevak C.C. (2010). Hyaluronan-CD44 Interaction Promotes c-Src-mediated Twist Signaling, MicroRNA-10b Expression, and RhoA/RhoC Up-regulation, Leading to Rho-kinase-associated Cytoskeleton Activation and Breast Tumor Cell Invasion. J. Biol. Chem..

[191] Gatti V., Fierro C., Compagnone M., Giangrazi F., Markert E.K., Bongiorno-Borbone L., Melino G., Peschiaroli A. (2018). DeltaNp63 regulates the expression of hyaluronic acid-related genes in breast cancer cells. Oncogenesis.

[192] Chanmee T., Ontong P., Izumikawa T., Higashide M., Mochizuki N., Chokchaitaweesuk C., Khansai M., Nakajima K., Kakizaki I., Kongtawelert P. (2016). Hyaluronan Production Regulates Metabolic and Cancer Stem-like Properties of Breast Cancer Cells via Hexosamine Biosynthetic Pathway-coupled HIF-1 Signaling. J. Biol. Chem..

[193] Zhang H., Fredericks T., Xiong G., Qi Y., Rychahou P.G., Li J., Pihlajaniemi T., Xu W., Xu R. (2018). Membrane associated collagen XIII promotes cancer metastasis and enhances anoikis resistance. Breast Cancer Res..

[194] Chen D., Bhat-Nakshatri P., Goswami C., Badve S.S., Nakshatri H. (2013). ANTXR1, a Stem Cell-Enriched Functional Biomarker, Connects Collagen Signaling to Cancer Stem-like Cells and Metastasis in Breast Cancer. Cancer Res..

[195] Liu C.-C., Lin S.-P., Hsu H.-S., Yang S.-H., Lin C.-H., Yang M.-H., Hung M.-C., Hung S.-C. (2016). Suspension survival mediated by PP2A-STAT3-Col XVII determines tumour initiation and metastasis in cancer stem cells. Nat. Commun..

[196] Yin P., Bai Y., Wang Z., Sun Y., Gao J., Na L., Zhang Z., Wang W., Zhao C. (2020). Non-canonical Fzd7 signaling contributes to breast cancer mesenchymal-like stemness involving Col6a1. Cell Commun. Signal..

[197] Tian J., Hachim M.Y., Hachim I.Y., Dai M., Lo C., Raffa F.A., Ali S., Lebrun J.J. (2017). Cyclooxygenase-2 regulates TGFbeta-induced cancer stemness in triple-negative breast cancer. Sci. Rep..

[198] Sun Y., Kim H.S., Saw P.E., Jon S., Moon W.K. (2015). Targeted Therapy for Breast Cancer Stem Cells by Liposomal Delivery of siRNA against Fibronectin EDB. Adv. Healthc. Mater..

[199] Chang C., Goel H.L., Gao H., Pursell B., Shultz L.D., Greiner D.L., Ingerpuu S., Patarroyo M., Cao S., Lim E. (2015). A laminin 511 matrix is regulated by TAZ and functions as the ligand for the alpha6Bbeta1 integrin to sustain breast cancer stem cells. Genes Dev..

[200] Cordenonsi M., Zanconato F., Azzolin L., Forcato M., Rosato A., Frasson C., Inui M., Montagner M., Parenti A.R., Poletti A. (2011). The Hippo Transducer TAZ Confers Cancer Stem Cell-Related Traits on Breast Cancer Cells. Cell.

[201] Berardi D.E., Raffo D., Todaro L.B., Simian M. (2017). Laminin Modulates the Stem Cell Population in LM05-E Murine Breast Cancer Cells through the Activation of the MAPK/ERK Pathway. Cancer Res. Treat..

[202] Lambert A.W., Wong C.K., Ozturk S., Papageorgis P., Raghunathan R., Alekseyev Y., Gower A.C., Reinhard B.M., Abdolmaleky H.M., Thiagalingam S. (2016). Tumor Cell-Derived Periostin Regulates Cytokines That Maintain Breast Cancer Stem Cells. Mol. Cancer Res..

[203] Oskarsson T., Acharyya S., Zhang X.H.-F., Vanharanta S., Tavazoie S.F., Morris P.G., Downey R.J., Manova-Todorova K., Brogi E., Massagué J. (2011). Breast cancer cells produce tenascin C as a metastatic niche component to colonize the lungs. Nat. Med..

[204] Insua-Rodríguez J., Pein M., Hongu T., Meier J., Descot A., Lowy C.M., De Braekeleer E., Sinn H., Spaich S., Sütterlin M. (2018). Stress signaling in breast cancer cells induces matrix components that promote chemoresistant metastasis. EMBO Mol. Med..

[205] Pio G.M., Xia Y., Piaseczny M.M., Chu J.E., Allan A.L. (2017). Soluble bone-derived osteopontin promotes migration and stem-like behavior of breast cancer cells. PLoS ONE.

[206] Xuejun T., Tian X., Oh S.Y., Movassaghi M., Naber S.P., Kuperwasser C., Buchsbaum R.J. (2016). The fibroblast Tiam1-osteopontin pathway modulates breast cancer invasion and metastasis. Breast Cancer Res..

[207] Sadr-Nabavi A., Ramser J., Volkmann J., Naehrig J., Wiesmann F., Betz B., Hellebrand H., Engert S., Seitz S., Kreutzfeld R. (2009). Decreased expression of angiogenesis antagonist EFEMP1 in sporadic breast cancer is caused by aberrant promoter methylation and points to an impact of EFEMP1 as molecular biomarker. Int. J. Cancer.

[208] Kwak J.-H., Lee N.-H., Lee H.-Y., Hong I.-S., Nam J.-S. (2016). HIF2α/EFEMP1 cascade mediates hypoxic effects on breast cancer stem cell hierarchy. Oncotarget.

[209] Hurt E.M., Chan K., Serrat M.A.D., Thomas S.B., Veenstra T.D., Farrar W.L. (2009). Identification of Vitronectin as an Extrinsic Inducer of Cancer Stem Cell Differentiation and Tumor Formation. Stem Cells.

[210] Neal J.T., Li X., Zhu J., Giangarra V., Grzeskowiak C.L., Ju J., Liu I.H., Chiou S.-H., Salahudeen A.A., Smith A.R. (2018). Organoid Modeling of the Tumor Immune Microenvironment. Cell.

[211] Jin M.-Z., Jin W.-L. (2020). The updated landscape of tumor microenvironment and drug repurposing. Signal Transduct. Target. Ther..

[212] Johnson K.E., Ceglowski J.R., Roweth H.G., Forward J.A., Tippy M.D., El-Husayni S., Kulenthirarajan R., Malloy M.W., Machlus K.R., Chen W.Y. (2019). Aspirin inhibits platelets from reprogramming breast tumor cells and promoting metastasis. Blood Adv..

[213] Hsieh C.-C., Wang C.-H. (2018). Aspirin Disrupts the Crosstalk of Angiogenic and Inflammatory Cytokines between 4T1 Breast Cancer Cells and Macrophages. Mediat. Inflamm..

[214] Saha S., Mukherjee S., Khan P., Kajal K., Mazumdar M., Manna A., Mukherjee S., De S., Jana D., Sarkar D.K. (2016). Aspirin Suppresses the Acquisition of Chemoresistance in Breast Cancer by Disrupting an NFkappaB-IL6 Signaling Axis Responsible for the Generation of Cancer Stem Cells. Cancer Res..

[215] Maity G., De A., Das A., Banerjee S., Sarkar S., Banerjee S.K. (2015). Aspirin blocks growth of breast tumor cells and tumor-initiating cells and induces reprogramming factors of mesenchymal to epithelial transition. Lab Investig..

[216] Khoo B.L., Grenci G., Lim J.S.Y., Lim Y.P., Fong J., Yeap W.H., Bin Lim S., Chua S.L., Wong S.C., Yap Y.-S. (2019). Low-dose anti-inflammatory combinatorial therapy reduced cancer stem cell formation in patient-derived preclinical models for tumour relapse prevention. Br. J. Cancer.

[217] Bhattacharya A., Mukherjee S., Khan P., Banerjee S., Dutta A., Banerjee N., Sengupta D., Basak U., Chakraborty S., Dutta A. (2020). SMAR1 repression by pluripotency factors and consequent chemoresistance in breast cancer stem-like cells is reversed by aspirin. Sci. Signal..

[218] Coyle C., Cafferty F.H., Rowley S., MacKenzie M., Berkman L., Gupta S., Pramesh C.S., Gilbert D., Kynaston H., Cameron D. (2016). ADD-ASPIRIN: A phase III, double-blind, placebo controlled, randomised trial assessing the effects of aspirin on disease recurrence and survival after primary therapy in common non-metastatic solid tumours. Contemp. Clin. Trials.

[219] Najafabadi A.H., Zhang J., Aikins M.E., Abadi Z.I.N., Liao F., Qin Y., Okeke E.B., Scheetz L.M., Nam J., Xu Y. (2020). Cancer Immunotherapy via Targeting Cancer Stem Cells Using Vaccine Nanodiscs. Nano Lett..

[220] Wang H., Chen N.G., Minev B., Szalay A.A. (2012). Oncolytic vaccinia virus GLV-1h68 strain shows enhanced replication in human breast cancer stem-like cells in comparison to breast cancer cells. J. Transl. Med..

[221] Ginestier C., Liu S., Diebel M.E., Korkaya H., Luo M., Brown M., Wicinski J., Cabaud O., Charafe-Jauffret E., Birnbaum D. (2010). CXCR1 blockade selectively targets human breast cancer stem cells in vitro and in xenografts. J. Clin. Investig..

[222] Doherty M.R., Cheon H., Junk D.J., Vinayak S., Varadan V., Telli M.L., Ford J.M., Stark G.R., Jackson M.W. (2017). Interferon-beta represses cancer stem cell properties in triple-negative breast cancer. Proc. Natl. Acad. Sci. USA.

[223] Visus C., Wang Y., Lozano-Leon A., Ferris R.L., Silver S., Szczepanski M.J., Brand R.E., Ferrone C.R., Whiteside T.L., Ferrone S. (2011). Targeting ALDH(bright) human carcinoma-initiating cells with ALDH1A1-specific CD8(+) T cells. Clin. Cancer Res..

[224] Anjanappa M., Cardoso A.A., Cheng L., Mohamad S., Gunawan A., Rice S., Dong Y., Li L., Sandusky G.E., Srour E.F. (2017). Individualized Breast Cancer Characterization through Single-Cell Analysis of Tumor and Adjacent Normal Cells. Cancer Res..

[225] Lawson D.A., Bhakta N.R., Kessenbrock K., Prummel K.D., Yu Y., Takai K., Zhou A., Eyob H., Balakrishnan S., Wang C.-Y. (2015). Single-cell analysis reveals a stem-cell program in human metastatic breast cancer cells. Nature.

[226] Yeo S.K., Zhu X., Okamoto T., Hao M., Wang C., Lu P., Lu L., Guan J.-L. (2020). Single-cell RNA-sequencing reveals distinct patterns of cell state heterogeneity in mouse models of breast cancer. eLife.

[227] Bartoschek M., Oskolkov N., Bocci M., Lövrot J., Larsson C., Sommarin M., Madsen C.D., Lindgren D., Pekar G., Karlsson G. (2018). Spatially and functionally distinct subclasses of breast cancer-associated fibroblasts revealed by single cell RNA sequencing. Nat. Commun..

[228] Azizi E., Carr A.J., Plitas G., Cornish A.E., Konopacki C., Prabhakaran S., Nainys J., Wu K., Kiseliovas V., Setty M. (2018). Faculty Opinions recommendation of Single-Cell Map of Diverse Immune Phenotypes in the Breast Tumor Microenvironment. Cell.

[229] Tekpli X., Osbreac, Lien T., Røssevold A.H., Nebdal D., Borgen E., Ohnstad H.O., Kyte J.A., Vallon-Christersson J., Fongaard M. (2019). An independent poor-prognosis subtype of breast cancer defined by a distinct tumor immune microenvironment. Nat. Commun..

